# Angiopoietin-like 3-derivative LNA043 for cartilage regeneration in osteoarthritis: a randomized phase 1 trial

**DOI:** 10.1038/s41591-022-02059-9

**Published:** 2022-12-01

**Authors:** Nicole Gerwin, Celeste Scotti, Christine Halleux, Mara Fornaro, Jimmy Elliott, Yunyu Zhang, Kristen Johnson, Jian Shi, Sandra Walter, Yufei Li, Carsten Jacobi, Nelly Laplanche, Magali Belaud, Jochen Paul, Gustavo Glowacki, Thomas Peters, Keith A. Wharton, Igor Vostiar, Florine Polus, Ina Kramer, Sabine Guth, Abdelkader Seroutou, Subhajit Choudhury, Didier Laurent, Joseph Gimbel, Jörg Goldhahn, Matthias Schieker, Sophie Brachat, Ronenn Roubenoff, Michaela Kneissel

**Affiliations:** 1 Novartis Institutes for BioMedical Research, Basel, Switzerland; 2 Novartis Pharma, Basel, Switzerland; 3 Novartis Institutes for BioMedical Research, San Diego, CA USA; 4 Novartis Institutes for BioMedical Research, Cambridge, MA USA; 5 Division of Scripps Research, Calibr, La Jolla, CA USA; 6 Praxisklinik Rennbahn, Muttenz, Switzerland; 7 Ultivue, Cambridge, MA USA; 8 IQVIA, Thane, India; 9 Arizona Research Center, Phoenix, AZ USA; 10 Institute for Translational Medicine, ETH Zürich, Zürich, Switzerland

**Keywords:** Osteoarthritis, Preclinical research

## Abstract

Osteoarthritis (OA) is a common, debilitating, chronic disease with no disease-modifying drug approved to date. We discovered LNA043—a derivative of angiopoietin-like 3 (ANGPTL3)—as a potent chondrogenesis inducer using a phenotypic screen with human mesenchymal stem cells. We show that LNA043 promotes chondrogenesis and cartilage matrix synthesis in vitro and regenerates hyaline articular cartilage in preclinical OA and cartilage injury models in vivo. LNA043 exerts at least part of these effects through binding to the fibronectin receptor, integrin α_5_β_1_ on mesenchymal stem cells and chondrocytes. In a first-in-human (phase 1), randomized, double-blinded, placebo-controlled, single ascending dose, single-center trial (NCT02491281; sponsored by Novartis Pharmaceuticals), 28 patients with knee OA were injected intra-articularly with LNA043 or placebo (3:1 ratio) either 2 h, 7 d or 21 d before total knee replacement. LNA043 met its primary safety endpoint and showed short serum pharmacokinetics, cartilage penetration and a lack of immunogenicity (secondary endpoints). Post-hoc transcriptomics profiling of cartilage revealed that a single LNA043 injection reverses the OA transcriptome signature over at least 21 d, inducing the expression of hyaline cartilage matrix components and anabolic signaling pathways, while suppressing mediators of OA progression. LNA043 is a novel disease-modifying OA drug candidate that is currently in a phase 2b trial (NCT04864392) in patients with knee OA.

## Abstract (web summary)

The mechanism of action of LNA043—a novel disease-modifying osteoarthritis treatment candidate—is characterized in preclinical studies and through transcriptomic profiling of cartilage from patients with osteoarthritis in a phase 1 trial.

## Main

Osteoarthritis (OA) is the most common degenerative joint disease, affecting more than 500 million people worldwide, and is a leading cause of disability among older adults^1,2^. OA most frequently affects knee joints. With no disease-modifying OA drug (DMOAD) available^3^, the only therapeutic options for knee OA are symptomatic treatments and, ultimately, joint replacement^4,5^.

A hallmark of OA pathogenesis is the progressive breakdown of articular cartilage, which is mediated by both enzymatic degradation of the cartilage matrix and deficient matrix formation^6^. On the cellular level, articular chondrocytes respond to the progressive accumulation of biochemical and biomechanical insults by shifting toward a degradative and hypertrophic state^7^. After cartilage injury, repair proceeds through fibrocartilage that has poor mechanical properties and eventually fails, leading to OA development rather than repaired hyaline cartilage^8^.

The goal of this study was to identify a DMOAD that regenerates hyaline cartilage through differentiation of endogenous mesenchymal stem cells (MSCs) into chondrocytes, which synthesize hyaline cartilage matrix. MSCs are capable of self-renewal and can differentiate into several cell lineages, including chondrocytes^9,10^. MSCs have been identified in both healthy and OA cartilage, as well as synovium and synovial fluid^10–15^, suggesting that the failure to regenerate healthy articular cartilage in OA may be due to impaired MSC regenerative activity and/or a hostile tissue environment rather than insufficient stem cell supply^16^.

To identify secreted proteins capable of selectively directing chondrogenic differentiation of primary human MSCs (hMSCs), an image-based high-throughput screen of a proprietary Novartis secretomics collection was performed, similar to that previously reported for small molecules^17^. The assay system modeled cartilage-resident MSCs to identify stimulators of their natural chondrogenesis potential and inducers of hyaline cartilage regeneration. Angiopoietin-like 3 (ANGPTL3) was identified as a hit from 6,300 screened candidate proteins, and its chondrogenic function was subsequently mapped to the carboxy (C)-terminal domain.

Here, we demonstrate that the novel ANGPTL3-derived protein LNA043 stimulates chondrogenic differentiation of endogenous MSCs and regenerates hyaline cartilage matrix in both in vitro and preclinical in vivo models. We show that LNA043 exerts at least part of this activity by binding to integrins. In a first-in-human (FIH) trial in patients with knee OA scheduled for total knee replacement (TKR), we demonstrate that intra-articular LNA043 is safe and induces prolonged reversion of the OA transcriptomics signature, stimulating the expression of hyaline cartilage matrix components and anabolic signaling pathways and suppressing known mediators of OA progression in the patient cartilage. Although the short treatment duration does not allow us to conclude that LNA043 provides clinical benefit, the data suggest that LNA043 is a promising candidate for the treatment of OA with a novel mechanism of action, warranting clinical development.

## Results

### LNA043 induces chondrogenesis and DKK1 secretion in vitro

**Fig. 1:**
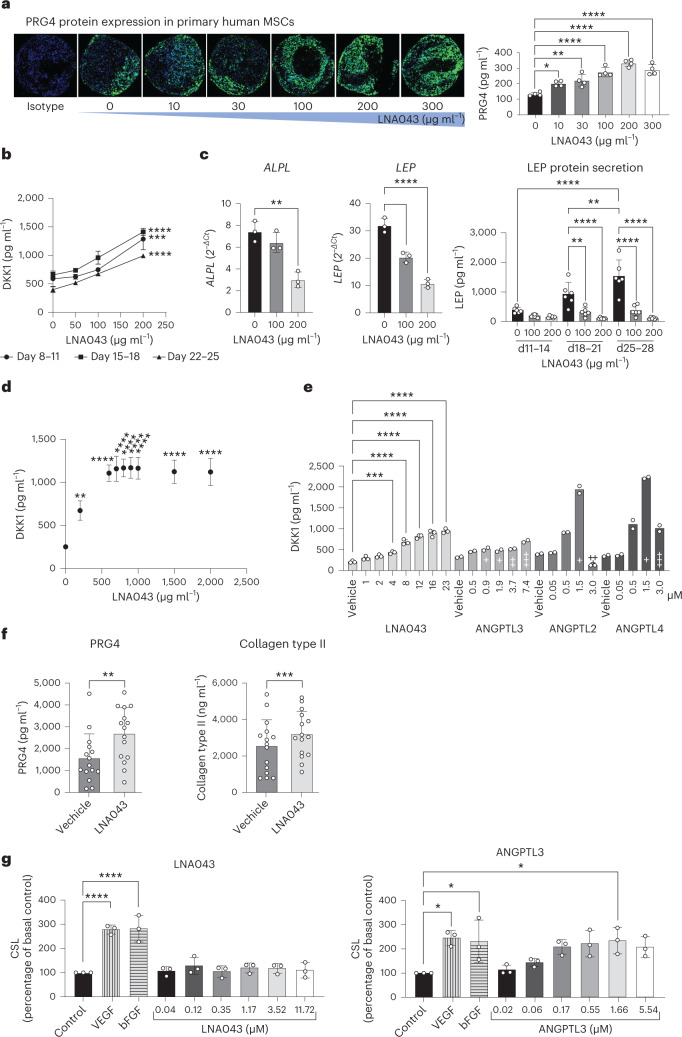
LNA043 induces chondrogenesis and DKK1, PRG4 and collagen type II secretion, but not angiogenesis. **a**, Left, PRG4 production by 3D hMSCs. PRG4 IHC staining after 28 d of LNA043 treatment. Green, PRG4; blue, DAPI. Comparable effects of LNA043 were detected in hMSCs from four (out of four) donors. Scale bar, 100 μm. Right, PRG4 secretion, as determined by ELISA, generated from one donor in quadruplicate (one representative experiment shown out of eight). **b**, DKK1 secretion by 3D hMSCs. The accumulation of DKK1 between days 8 and 11, 15 and 18 and 22 and 25 was quantified by ELISA (one representative experiment shown out of three; *n* = 3 biological replicates). Comparable effects of LNA043 were detected in hMSCs from two (out of four) donors. **c**, Osteogenic and adipogenic marker expression in 3D hMSCs. *ALPL* (left) and *LEP* expression (middle) were determined by quantitative reverse transcription PCR (one representative experiment shown out of six; *n* = 3 biological replicates). Right, LEP secretion as determined by ELISA (one representative experiment shown out of four; *n* = 6 biological replicates) following 28 d of LNA043 treatment. Comparable LNA043 effects on *ALPL*, *LEP* and LEP levels were detected in hMSCs from four (out of four) donors. **d**, DKK1 secretion by C-28/I2 cells, as determined by ELISA, following 24 h culture with LNA043 (one representative experiment shown out of >10; *n* = 3 biological replicates). **e**, DKK1 secr**e**tion by C-28/I2 cells, as determined by ELISA, following 24 h culture in the presence of LNA043 (23 μM = ~600 μg ml^−1^), ANGPTL3, ANGPTL2 or ANGPTL4 one representative experiment shown out of two; *n* = 2–3 biological replicates). Single, double and triple crosses represent the first signs of toxicity, few intact cells remaining and many particles or cell debris present, respectively. The toxicity scores are based on cell morphology (bright-field microscopy). **f**, PRG4 (left) and collagen type II increase (right) in the supernatants of human OA cartilage explants, as determined by ELISA (*n* = 15; from two donors). One half of each explant was cultured with LNA043, while the other half was cultured with vehicle for 24 h. **g**, LNA043 (left), unlike ANGPTL3 (right), does not induce angiogenesis. The cumulative sprout length (CSL) was measured in HUVEC spheroids incubated with LNA043, ANGPTL3, VEGF-A (25 ng ml^−1^) or bFGF (25 ng ml^−1^) for 24 h. The data are representative of three independent experiments. Each individual data point per experiment represents the mean CSL of ten randomly selected spheroids and is expressed as a percentage of basal control. In **a**–**g**, the values represent means ± s.d. Statistical significance was determined by one-way ANOVA (**a**, **b**, **d**, **e** and **g**), one-way ANOVA performed on each day separately (**c**) or two-tailed paired *t*-test (**f**). *P* values are provided in Extended Data Table 1 (*P < 0.05; **P < 0.01; ***P < 0.001; ****P < 0.0001).

LNA043 is a 26 kDa protein comprising the C-terminal fibrinogen-like (FBN-like) domain of ANGPTL3 and a single point mutation to prevent proteolysis during recombinant protein production (Supplementary Fig. 1). LNA043 maintains the chondrogenic potential of ANGPTL3, as demonstrated by its ability to induce cartilage anabolic marker expression in a three-dimensional (3D) primary hMSC chondrogenesis assay. LNA043 treatment over 28 d induced a dose-dependent increase (up to 2.5-fold) of the cartilage superficial zone glycoprotein lubricin/proteoglycan 4 (PRG4)^18^, as detected by immunohistochemistry (IHC) staining of the 3D pellets and by enzyme-linked immunosorbent assay (ELISA) in supernatants (Fig. 1a and Extended Data Table 1). Furthermore, LNA043 dose dependently upregulated secretion of the wingless and int-related protein (WNT) inhibitor protein dickkopf-1 (DKK1)^19^ in supernatants over the 25 d culture period (Fig. 1b). While LNA043 did not induce gene expression of *COL2*, *ACAN* or *COL10*, it decreased the osteogenesis marker alkaline phosphatase (*ALPL*)^20^ and the pro-inflammatory adipokine leptin (*LEP*)^21^ both at the transcript and protein levels (Fig. 1c).

**Extended Data Fig. 1:**
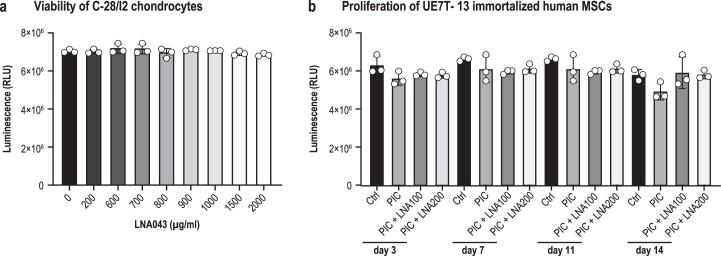
Viability of C-28/I2 cells upon LNA043 treatment and absence of a proliferative effect of LNA043 during chondrogenic rescue in UE7T-13 cells. **a**, Viability of C-28/I2 cells upon LNA043 treatment. Absence of cytotoxicity of LNA043 in C-28/I2 cells treated for 24 h in C-28/I2 test medium supplemented with LNA043 was determined by CellTiter-Glo® (Promega). Levels are expressed in relative light units (RLU). Values are mean ± s.d. of measurements from a representative experiment with biological triplicates. **b**, Absence of a proliferative effect of LNA043 during the chondrogenic rescue in UE7T-13 cells. Proliferation was assessed in UE7T-13 cells cultivated for 3, 7, 11 and 14 d in UE7T-13 chondrogenic medium supplemented or not with 0.5 μg ml^-1^ Poly I:C (PIC), 100 or 200 μg ml^-1^ LNA043 by CellTiter-Glo® (Promega). Levels are expressed in relative light units (RLU). Values are mean ± s.d. of measurements from biological triplicates. In **a** and **b** statistical significance was determined by one-way ANOVA. No significant differences were observed between any of the conditions.

We then tested LNA043 on mature chondrocytes using the human immortalized chondrocyte cell line C-28/I2. Consistent with the effects on MSCs, a rapid (24 h), dose-dependent increase of secreted DKK1 protein was observed (up to 4.5-fold; Fig. 1d). LNA043 did not induce cytotoxicity at concentrations as high as 2 mg ml^−1^ (Extended Data Fig. 1a). ANGPTL3, and to an even greater extent ANGPTL2 and ANGPTL4 (previously reported to be expressed in cartilage and able to induce cartilage erosion^22^ and chondrogenesis^23^, respectively), stimulated DKK1 secretion more potently (up to 20-fold) than LNA043. However, in contrast with LNA043, they also induced strong cytotoxicity at concentrations as low as 3 μM (Fig. 1e).

To study the effects of LNA043 on human OA cartilage we leveraged an ex vivo explant assay. Full-thickness cartilage plugs from tibia plateaus were collected at TKR, selecting areas with various degrees of OA-related cartilage damage. Interestingly, supernatants of the LNA043-treated explants contained significantly higher amounts of the key cartilage components collagen type II and PRG4 than those of vehicle-treated controls (Fig. 1f).

Since the C-terminal domain of ANGPTL3 has been reported to induce angiogenesis in vivo^24^, we assessed the potential risk of blood vessel formation by LNA043 versus ANGPTL3 in an in vitro human umbilical vein endothelial cell (HUVEC) sprouting assay^25^. While ANGPTL3 at 0.02–5.5 μM concentrations induced angiogenesis with a sprouting length comparable to those of vascular endothelial growth factor (VEGF) and basic fibroblast growth factor (bFGF) controls, no sprouting was observed with 0.04–11.7 μM LNA043 (Fig. 1g), suggesting that LNA043 does not induce angiogenesis.

### LNA043 partly preserves chondrogenesis under inflammatory conditions

**Extended Data Fig. 2:**
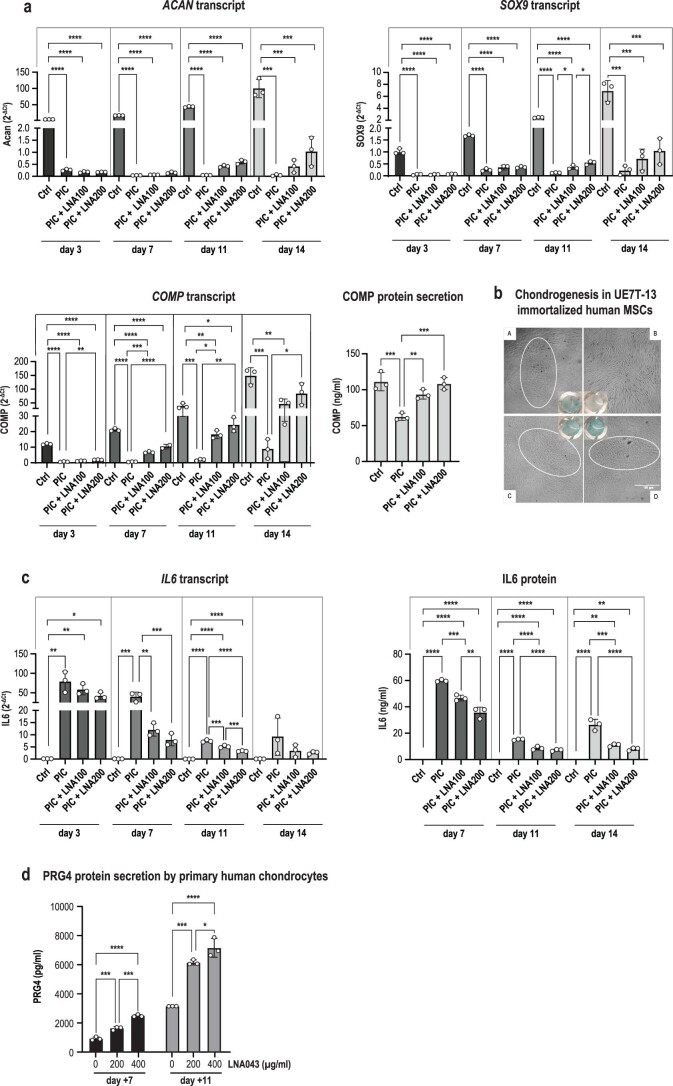
LNA043 partially preserves chondrogenic activity in immortalized hMSC and primary human OA chondrocytes in inflammatory conditions. **a-d**, UE7T-13 cells cultivated in chondrogenic medium supplemented with Poly I:C (PIC) and/or LNA043 (100 or 200 μg ml^-1^) as indicated. **a**, *ACAN, SOX9* and *COMP* gene expression after 3, 7, 11 and 14 d culture. COMP secretion (ELISA) following 14 d treatment; one representative experiment is shown out of three, with n=3 biological replicates. **b**, Phase-contrast microscopic appearance of representative UE7T-13 cells cultured for 11 d in chondrogenic medium (A) or supplemented with 0.5 μg ml^-1^ Poly I:C (B) or 0.5 μg ml^-1^ Poly I:C and 100 μg ml^-1^ LNA043 (C) or 0.5 μg ml^-1^ Poly I:C and 200 μg ml^-1^ LNA043 (D). Areas of cartilage nodule formation are encircled by lines; scale bar = 100 μm. Middle: Alcian blue staining at 10 d in a 96-well plate in chondrogenic medium supplemented as indicated in A, B, C and D; one representative experiment shown out of three. **c**, IL6 gene and protein expression after 3 (gene only), 7, 11 and 14 d culture; one representative experiment shown out of three, with n=3 biological replicates. **d**, PRG4 ELISA of supernatant from 3D cultures of primary human OA articular chondrocytes, originating from one donor (one representative experiment shown out of three, n=3 biological replicates). After 14 d in chondrogenic medium cells were treated for 3 d with TNFα + IL-1β followed by a 7 and 11 d recovery period in the presence of LNA043 or vehicle. Comparable effects of LNA043 were detected with primary OA chondrocytes from 3 different donors. **a, c, d**, Values are means ± s.d. **a, c, d**, Statistical significance was determined by one-way ANOVA performed on each day separately; *P-*values are provided in Extended Data Table 2. (*P < 0.05; **P < 0.01; ***P < 0.001; ****P < 0.0001).

To assess whether LNA043 remains active in an inflammatory OA environment, we evaluated the expression of the chondrogenic markers aggrecan (*ACAN*), cartilage oligomeric matrix protein (*COMP*) and SRY-box transcription factor 9 (*SOX9*) in an immortalized human UE7T-13 MSC differentiation assay in the presence or absence of inflammatory insult polyinosinic–polycytidilic acid (poly(I:C))^26^. *ACAN*, *COMP* and *SOX9* transcripts increased over the course of differentiation under control chondrogenic conditions as expected. Poly(I:C) treatment sharply decreased the expression of these three chondroanabolic markers (Extended Data Fig. 2a and Extended Data Table 2) and resulted in the disappearance of chondrogenic nodules and Alcian blue staining of cartilage glycosaminoglycans (Extended Data Fig. 2b). Simultaneous treatment with LNA043 partially preserved chondrogenesis, as indicated by sustained chondrogenic marker gene expression in a LNA043 dose- and treatment time-dependent fashion (that is, *ACAN* expression was maintained at detectable levels, while *COMP* and *SOX9* transcript levels were kept at 65 and 26% of chondrogenesis control levels, respectively). The LNA043 effect was even stronger at the protein level, as COMP protein secretion was almost fully preserved (97%; Extended Data Fig. 2b). In addition, concomitant LNA043 treatment inhibited the poly(I:C)-induced expression of interleukin-6 (IL-6) in a dose- and time-dependent manner by 80% at the transcript level and 71% at the protein level (Extended Data Fig. 2c). LNA043 did not induce UE7T-13 cell proliferation during the chondrogenic rescue (Extended Data Fig. 1b).

Next, we investigated whether LNA043 also induces cartilage regeneration in human OA chondrocytes after inflammatory stimulation. To this end, primary human articular chondrocytes collected at TKR were re-differentiated in 3D cultures, followed by stimulation with the inflammatory cytokines tumor necrosis factor-α (TNF-α) + IL-1β. Subsequent treatment with LNA043, but not vehicle, restored PRG4 secretion in a dose- and time-dependent manner (Extended Data Fig. 2d). Taken together, the data suggest that LNA043 preserved chondrogenic activity at least partially after inflammatory stimulation.

### Integrin α_5_β_1_ is required for DKK1 secretion and binds LNA043

**Fig. 2:**
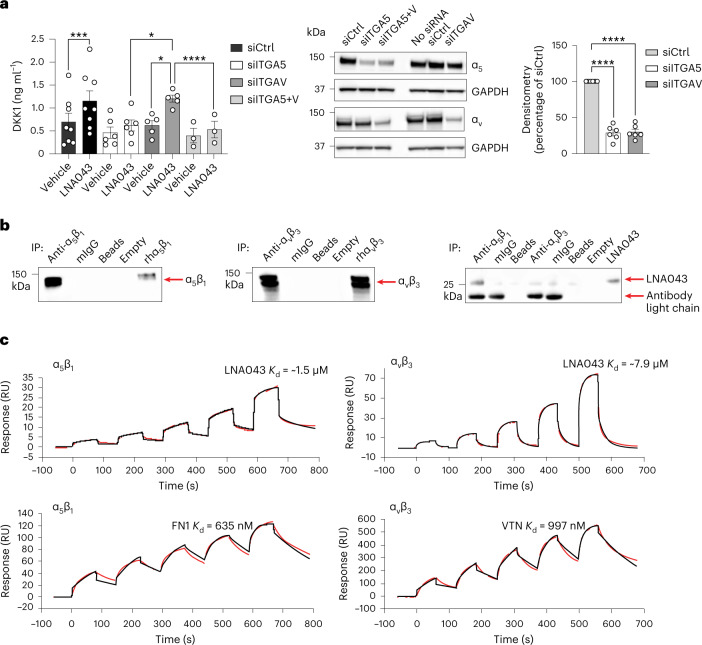
Integrin α_5_β_1_ is required for LNA043-induced DKK1 secretion by C-28/I2 chondrocytes and binds LNA043. **a**, LNA043-induced DKK1 secretion is integrin α_5_-dependent. Integrins α_5_ and α_v_ were knocked down in C-28/I2 cells by siRNA (siITGA5 and siITGAV, respectively; siITGA5+V for both combined). Left, DKK1 secretion induced by LNA043 (200 μg ml^−1^), as analyzed by ELISA. The data represent means ± s.e.m. of 3–8 independent experiments (from left to right, ***P = 0.0003, *P = 0.0391, *P = 0.0178 and ****P ≤ 0.0001). Statistical significance was determined by one-way ANOVA with mixed-effect analysis and Šídák’s multiple comparison test. Middle, efficiency of the knockdown, as monitored by immunoblotting. GAPDH was used as a loading control. Right, quantification of the immune bands, as determined by densitometry. The data are presented as a percentage decrease ± s.e.m. versus control siRNA (siCtrl) and represent six independent experiments (****P ≤ 0.0001). Statistical significance was determined by one-way ANOVA with Tukey’s multiple comparison test. **b**, LNA043 interacts with α_5_β_1_ integrin in vitro, as shown by co-IP experimentation. Recombinant human α_5_β_1_ integrin (rhα_5_β_1_) or recombinant human α_v_β_3_ integrin (rhα_v_β_3_) were incubated with LNA043 for 1 h at 37 °C. Immunoprecipitation was performed using a monoclonal antibody to α_5_β_1_ (anti-α_5_β_1_) or α_v_β_3_ integrin (anti-α_v_β_3_) followed by western blot analysis (right) with monoclonal antibodies to the α_5_ (left) or α_v_ (middle) integrin subunit or ANGPTL3, which recognize LNA043 (right). One representative experiment is shown out of two to four independent experiments. **c**, SPR single-cycle binding studies. LNA043 (top left and top right), FN1 (bottom left) or VTN (bottom right) were immobilized on a CM5 sensor. Integrins α_5_β_1_ (left) or α_v_β_3_ (right) served as analytes (0.6–10 µM). The sensorgrams were fitted with a 1:1 binding fit. One representative experiment is shown out of three independent experiments with comparable results. RU, resonance units. Source data

We hypothesized that integrins α_v_β_3_ and α_5_β_1_ may play a key role in the mechanism of action of LNA043, since the ANGPTL3 C-terminal domain binds integrin α_v_β_3_^24^, the vitronectin (VTN) receptor, and our LNA043 transcriptomics analyses (see below) suggested that fibronectin (FN1), the ligand of integrin α_5_β_1_, may be a central interaction partner of LNA043-regulated OA genes. Furthermore, interaction of the cartilage extracellular matrix (ECM) proteins FN1 and VTN with integrins expressed on chondrocytes is known to regulate cell differentiation and survival^27^. Therefore, we explored whether the LNA043-induced secretion of DKK1 by chondrocytes was integrindependent by knocking down integrin subunits α_v_, α_5_ or both in C-28/I2 cells using small interfering RNA (siRNA). Knockdown efficiency was confirmed by western blotting (Fig. 2a). Interestingly, silencing of α_5_, but not of α_v_, significantly inhibited LNA043-induced DKK1 secretion, and knockdown of both integrins did not result in further inhibition (Fig. 2a). This suggested that the FN1 receptor, integrin α_5_β_1_, but not the previously identified interaction partner of ANGPTL3,the VTN receptor integrin α_v_β_3_, is required for LNA043 activity. Co-immunoprecipitation (co-IP) experiments revealed the presence of LNA043 in co-IPs with α_5_β_1_, but not α_V_β_3_ or mouse immunoglobulin G (mIgG), suggesting that LNA043 interacts directly with integrin α_5_β_1_, but not α_v_β_3_ (Fig. 2b).

Next, we explored the binding of integrin α_5_β_1_ to LNA043 and FN1 and the binding of integrin α_v_β_3_ to LNA043 and VTN using surface plasmon resonance (SPR). The mean *K*_d_ values for α_5_β_1_–FN1 and α_v_β_3_–VTN interactions were in the nM range (635 and 997 nM, respectively), while integrin α_5_β_1_ and α_v_β_3_ binding to LNA043 was observed only in the low µM range (means of approximately 1.5 and 7.9 μM, respectively). Furthermore, the binding profile of the integrins to LNA043 did not follow a pure 1:1 binding fit, probably due to the conformational heterogeneity of the recombinant integrin molecules observed in cryogenic electron microscopy, which did not allow an accurate *K*_d_ determination (Fig. 2c). These results suggest that LNA043 can bind to recombinant integrins α_5_β_1_ and α_v_β_3_ under SPR conditions.

### LNA043 regenerates cartilage in OA and cartilage injury models

**Fig. 3:**
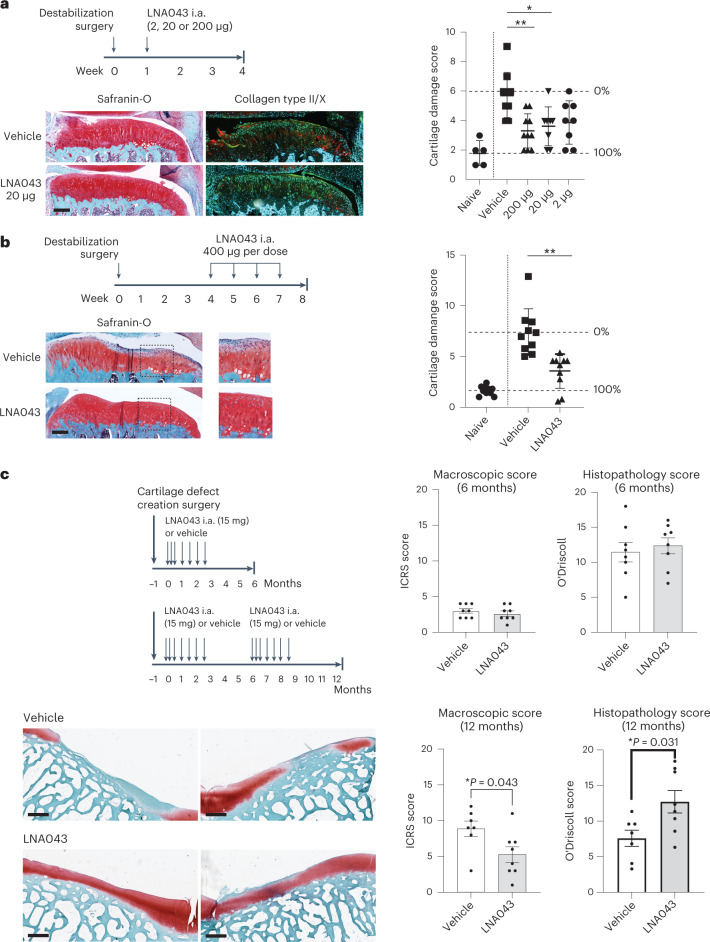
LNA043 treatment regenerates cartilage in rat OA and minipig cartilage injury models. **a**, Short-term rat OA model. Top left, schematic of the experimental design. i.a., intra-articular. Bottom left, representative images of knee joint sections from rats treated with vehicle or 20 μg LNA043 and stained with Safranin-O (red) or combined collagen type II (green) and X (red) IHC and counterstained with DAPI at 4 weeks post-RMT surgery. Scale bar, 200 μm. Right, cartilage damage, as determined by modified OARSI scoring (Supplementary Table 4) for naive (no surgery; *n* = 5), vehicle-treated (*n* = 9) and LNA043-treated rats (for 2 μg, *n* = 8; for 20 μg, *n* = 8; for 200 μg, *n* = 10). The data represent means ± s.d. Each data point represents the results from an individual rat. The percentage cartilage repair in LNA043-treated rats was calculated as the improvement in the score in vehicle-treated (0%) compared with naive rats (100%). Statistical significance was determined by Kruskal–Wallis non-parametric test with Dunn’s multiple comparison (*P = 0.279; **P = 0.0041). **b**, Therapeutic rat OA model. Top left, schematic of the experimental design. Bottom left, representative images of knee joint sections from rats treated with four weekly injections of vehicle or 400 μg LNA043 and stained with Safranin-O (red) 8 weeks post-meniscal tear surgery. Scale bar, 200 μm. The images to the right are magnifications of the sections highlighted by a dashed box to the left. Right, cartilage damage, as determined by modified OARSI scoring for naive, vehicle-treated and LNA043-treated rats (*n* = 10 per group). Data analysis and representation as in **a** (**P ≤ 0.0027). For **a** and **b**, the maximum cartilage damage score was 16. **c**, Minipig cartilage injury model. Top left, schematic of the experimental design. Bottom left, representative images of cartilage defect sites in trochlea stained with Safranin-O (red) at 12 months after two cycles of seven weekly/biweekly intra-articular injections of vehicle or 15 mg LNA043. The left and right images show the results from two different pigs. Scale bars, 700 μm. Right, cartilage quality was assessed macroscopically by the ICRS cartilage damage score (top and bottom left; Supplementary Table 5; 0 = normal; 20 = maximum damage), and cartilage repair was quantified in histopathology sections using a modified O’Driscoll cartilage repair score (top and bottom right; Supplementary Table 6; 21 = maximum repair), for vehicle-treated (*n* = 8) and LNA043-treated minipigs (*n* = 8) at 6 months (top) and vehicle-treated (*n* = 7) and LNA043-treated minipigs (*n* = 8) at 12 months (bottom). The data represent means ± s.e.m. Statistical significance was determined by two-tailed non-parametric Mann–Whitney *U*-test.

To evaluate whether in vitro LNA043 chondrogenesis activity translates into cartilage regeneration in vivo, we first tested the ability of LNA043 to reduce cartilage degeneration in a short-term rat meniscal tear (RMT) model of post-traumatic early OA^28^, which is characterized by mild cartilage degeneration with proteoglycan loss, surface irregularities, fissures, fibrillations, cysts and cell loss at 4 weeks post-surgery. A single intra-articular injection 1 week after surgical OA induction, when cartilage damage started to develop, decreased the modified Osteoarthritis Research Society International (OARSI) cartilage degeneration histopathology score^28^ by 48% at 2 μg per knee and up to 62% at 200 μg per knee (Fig. 3a). Since spontaneous or surgical cartilage repair is characterized by default replacement of hyaline articular cartilage with low-quality, short-lived fibrocartilage^8^, we assessed cartilage quality using IHC staining. A single intra-articular injection of 20 μg LNA043 decreased hypertrophic cartilage marker collagen type X and increased hyaline cartilage marker collagen type II, suggesting the preservation and regeneration of healthy hyaline cartilage with LNA043 treatment (Fig. 3a).

Next, we evaluated the cartilage repair potential of LNA043 in a therapeutic mode. Early OA was allowed to develop over 4 weeks after RMT surgery, before starting weekly intra-articular treatment with 400 μg LNA043 or vehicle for 4 weeks. At 8 weeks post-surgery, OA development had progressed to proteoglycan loss in deep zones, formation of cysts, cartilage damage and loss in superficial as well as deep zones; occasional full-thickness lesions were present in the medial tibial plateau of vehicle-treated joints (Fig. 3b). LNA043 treatment improved cartilage integrity and proteoglycan content and decreased the cartilage degeneration score by 67% compared with vehicle (Fig. 3b), suggesting that LNA043 not only prevents but also repairs cartilage damage in preclinical in vivo models of OA.

To investigate the sustainability of LNA043-induced hyaline repair cartilage, we surgically created a large cartilage defect in the trochlea of minipigs^29^ and evaluated cartilage repair macroscopically and histologically after one and two treatment cycles of seven weekly/biweekly intra-articular injections of 15 mg LNA043 or vehicle at 6 and 12 months, respectively (Fig. 3c). A single treatment cycle resulted in some filling of the defect with fibrocartilage in both LNA043- and vehicle-treated minipigs at 6 months and therefore similar O’Driscoll histopathology cartilage repair scores (Fig. 3c). At 12 months, two cycles of LNA043 treatment resulted in improved macroscopic cartilage defect filling, as determined by International Cartilage Repair Society (ICRS) cartilage damage scoring^30^, as well as increased amounts of healthy hyaline cartilage compared with vehicle in O’Driscoll cartilage repair scoring^31^ (Fig. 3c).

In summary, the data suggest that LNA043 both preserves and regenerates hyaline cartilage, which is stable over a prolonged period in preclinical models of cartilage injury and OA. Importantly, there were no adverse events associated with LNA043 treatment in preclinical OA models or follow-up toxicology studies, nor was cartilage overgrowth or ectopic cartilage formation observed, suggesting that LNA043 treatment has the potential to be safe and efficacious in patients with OA.

### LNA043 is safe and well tolerated in patients with knee OA

**Fig. 4:**
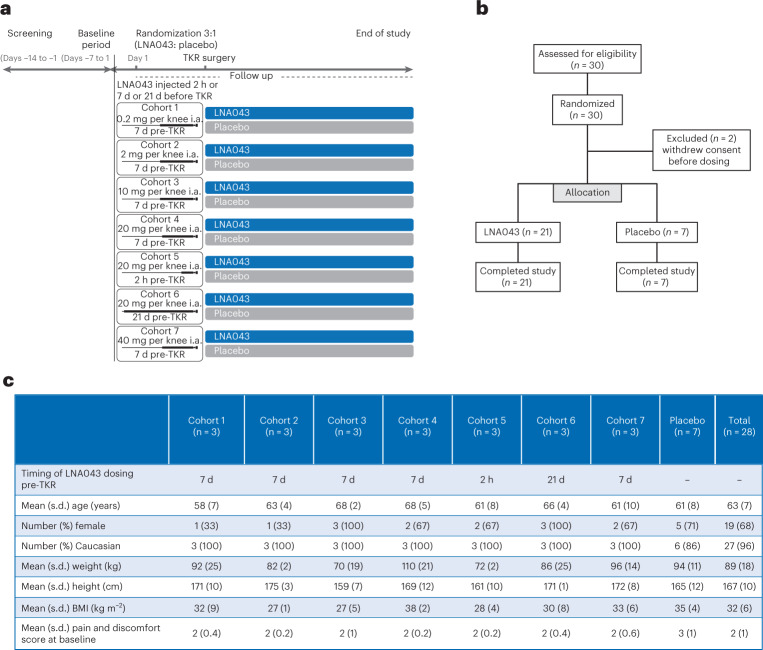
LNA043 FIH study design and baseline data. **a**, CLNA043X2101 study design summarizing the study periods and treatment arms. The end of study visit was at day 36 for cohorts 1–4 and 7, at day 29 for cohort 5 and at day 50 for cohort 6. **b**, CONSORT diagram showing the flow of patients at each stage of the FIH trial. **c**, Baseline data and demographics. BMI, body mass index.

**Fig. 5:**
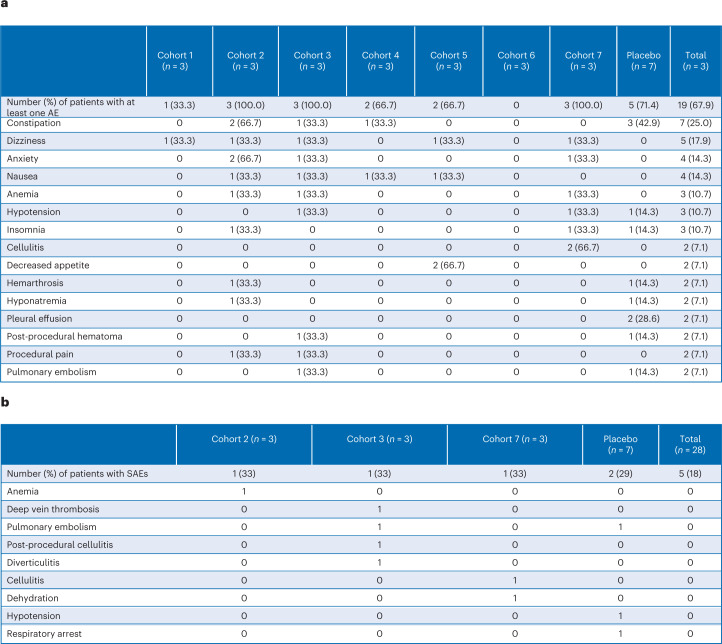
LNA043 is safe in the FIH study. **a**, Table summarizing the adverse events (AEs) that occurred at least twice, reported per treatment arm and not showing clear trends in safety. **b**, Table reporting all of the serious adverse events (SAEs), also not showing safety trends, and with none of them suspected to be related to LNA043. Cohorts 1 and 4–6 are not included as there were no SAEs in these cohorts; however, the final column includes these participants in the total.

A FIH, randomized, single-center, placebo-controlled, double-blind, single ascending dose trial (NCT02491281) was conducted in male (*n* = 9; 32%) and female (*n* = 19; 68%) patients with OA who were scheduled for TKR. Patients were randomized (LNA043:placebo = 3:1) to receive one of five increasing intra-articular dose levels (0.2, 2, 10, 20 or 40 mg; four patients per cohort) administered 7 d before TKR. The 20 mg dose was also administered 2 h or 21 d before TKR (Fig. 4a,b). Baseline values and demographics were comparable between cohorts (Fig. 4c). Overall, LNA043 was safe and well tolerated. Nineteen patients (14 on LNA043 and 5 on placebo) experienced at least one adverse event. The overall incidence of adverse events with LNA043 was 66.7% (14/21); with placebo, it was 71.4% (5/7). These were generally due to the TKR. No clinically relevant drug-related adverse events were reported (Fig. 5a), besides one patient receiving 40 mg LNA043 reporting two adverse events of transient dry mouth and dysgeusia (metallic taste in mouth), which were mild in severity and suspected to be drug related. Both adverse events resolved before the end of the study. Most of the adverse events reported were mild (37 in 14 patients (50%)) or moderate (44 in 12 patients (43%)). Twelve severe adverse events were reported in six patients (21%). None of these severe adverse events were suspected to be drug related. Ten serious adverse events were reported in five patients, none of which were suspected by the investigator to be related to LNA043 (Fig. 5b). No anti-LNA043 antibodies were detected. Safety laboratory evaluations, as well as vital signs and the Knee injury and Osteoarthritis Outcome Score (KOOS; http://www.koos.nu/koos-english.pdf), did not reveal any drug-related safety concerns.

### LNA043 is only transiently detectable in serum

**Extended Data Fig. 3:**
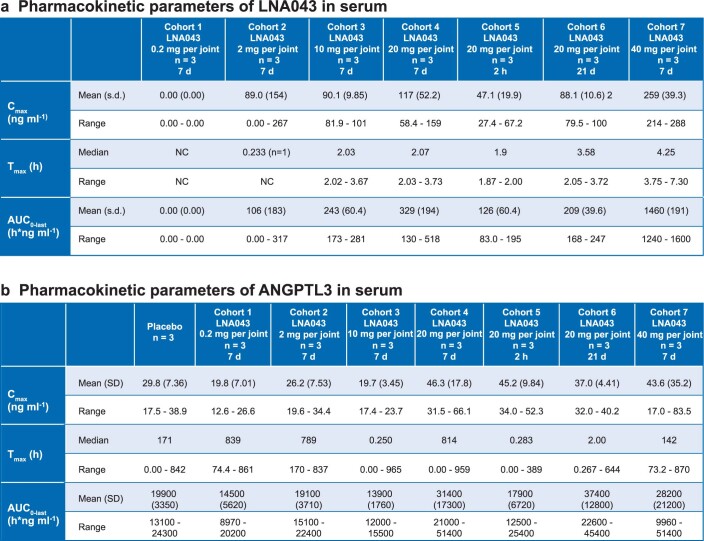
Tables summarizing the key pharmacokinetic parameters. **a**, LNA043 pharmacokinetics, demonstrating fast clearance of LNA043 from circulation, and **b**, ANGPTL3 pharmacokinetics, not showing any impact of exposure to LNA043.

LNA043 was detectable in the serum (>10 ng ml^−1^) of patients receiving 10 and 20 mg up to 4 h post-dose and receiving 40 mg up to 8 h post-dose, while only one of three patients receiving 2 mg and none receiving 0.2 mg had detectable serum levels. Thus, LNA043 rapidly distributed from the joint to the circulation. The serum exposures showed a high degree of inter-subject variability but tended to increase with increasing dose (Extended Data Fig. 3a). ANGPTL3 serum concentration profiles did not change after the administration of LNA043 (Extended Data Fig. 3b) and were within the expected range of variability^32^. In the majority of synovial fluid samples, ANGPTL3 and LNA043 were not quantifiable (lower limit of quantitation < 2.74 and <20 ng ml^−1^, respectively).

### LNA043 penetrates human OA cartilage

**Fig. 6:**
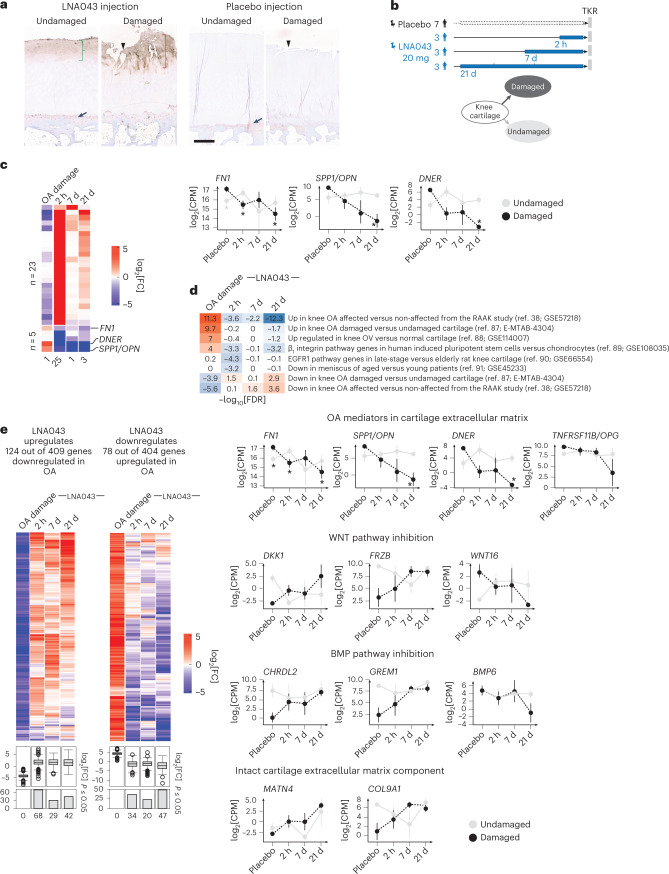
LNA043 penetrates damaged cartilage and reverts the OA transcriptional signature in FIH study patients. **a**, Representative images of LNA043 detection by IHC in TKR cartilage from patients with OA in the FIH trial 2 h post-LNA043 injection (left) or after placebo treatment (right). LNA043 (brown stain) showed deeper penetration into damaged (red bracket) than intact cartilage (green bracket). Cartilage from placebo-injected patients showed no LNA043-specific staining. Unspecific staining was seen in all specimens at the tidemark (arrows). The arrowheads indicate the superficial tear characteristic of damaged cartilage. These images are representative of 21 (LNA043 injection) and 49 (placebo treatment) IHC-stained cartilage samples. Scale bar, 600 μm. **b**, Transcriptomics study design. Damaged and undamaged articular cartilage samples were profiled from patients with OA injected with placebo (*n* = 7) or 20 mg LNA043 at 2 h, 7 d or 21 d (*n* = 3 each) before TKR. **c**, Left, heatmap showing the expression changes expressed as log_2_[fold change] (log_2_[FC]) of the 28 genes statistically significantly regulated by LNA043. OA damage refers to damaged versus undamaged cartilage in the placebo group; 2 h, 7 d and 21 d refer to damaged cartilage with LNA043 compared with placebo treatment. Numbers below the heatmap indicate the number of significantly regulated genes for each timepoint comparison. The 23 genes at the top are upregulated by LNA043, and the 5 genes at the bottom are downregulated by LNA043. Right, temporal expression profiles of three genes significantly and consistently downregulated by LNA043, expressed as log_2_[counts per million reads] (log_2_[CPM]). Statistical significance was determined by limma moderated *t*-test (defined as a Benjamini–Hochberg adjusted *P* value of ≤0.05 versus placebo; *P_*FN1*_ = 8.40 × 10^−5^ (2 h); *P_*FN1*_1.57 × 10^−9^ (21 d); *P_*SPP1/OPN*_ = 1.09 × 10^−4^; *P_*DNER*_ = 0.012145). The data are presented as means ± s.d. **d**, Significant concordance between curated public OA disease signatures and FIH-derived OA signatures. LNA043 gene signatures inversely correlate with OA disease signatures. These were generated from the SkeletalVis repository and from published transcriptomics studies. Enrichment is expressed as the negative log_10_ of the false discovery rate [FDR] from GSEA. The signs indicate the direction of enrichment. Blue color intensity indicates enrichment in downregulated genes, and red color enrichment in upregulated genes. EGFR1, epidermal growth factor receptor 1. **e**, Reversal of OA-associated transcriptome changes in the FIH study by LNA043. Left, heatmaps of expression changes (log_2_[FC]) for genes regulated with a nominal limma moderated *t*-test *P* ≤ 0.05. Below the heatmaps are boxplots summarizing the log_2_[FC] for all genes in the heatmap per group. Central lines and box edges represent median values and upper and lower quartiles, respectively, and the whiskers represent 1.5× the interquartile range. The numbers of genes counter-regulated by LNA043 are shown in the bar plots below the boxplots. Right, temporal transcriptional response to LNA043 for OA-relevant pathway genes (expressed as the mean log_2_[CPM] ± s.d.). Statistical significance was defined as in **c**.

**Extended Data Fig. 4:**
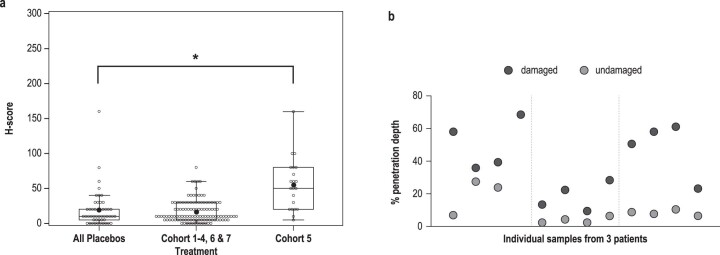
LNA043 cartilage penetration in the FIH study. Data are analyzed using a linear mixed-effect model with repeated measures. The two-sided *P*-value was obtained by *t*-statistic. **a**, LNA043 staining detected only in cohort 5 samples from the CLNA043X2101 study, when LNA043 was injected 2 h before TKR. Individual data points and boxplot of the LNA043 staining score of osteochondral samples from all cohorts are shown. Positive staining was observed in cohort 5 (*P = 0.0002). Boxplot data is expressed as median and lower/upper quartiles and whiskers at 1.5 times the interquartile range. **b**, LNA043 diffuses deeper into damaged compared to undamaged cartilage. Scatter plot of LNA043 penetration depth in cartilage samples from cohort 5, expressed as % of cartilage thickness, which is positively stained. Measurements were performed by an expert pathologist (KAW) on n=11 samples, with 4 samples harvested from different areas of the knee (osteochondral biopsies from femoral condyle, patella, trochlea, tibia) for each of the 3 patients (1 sample missing, because there was no cartilage left). Dotted lines separate data from individual patients. *P* = 0.000000475 for LNA043 penetration depth in damaged compared to undamaged cartilage.

Consistent with these pharmacokinetic data, LNA043 was not detectable by IHC in articular cartilage or soft tissue samples 7 or 21 d after administration, but was present in superficial articular cartilage and soft tissues in all patients who received LNA043 2 h before TKR. A post-hoc analysis showed that LNA043 diffused 4-fold deeper into damaged compared with undamaged cartilage within the same joint (Fig. 6a and Extended Data Fig. 4a,b).

### LNA043 reverses the OA transcriptional signature in patients with OA

**Extended Data Fig. 5:**
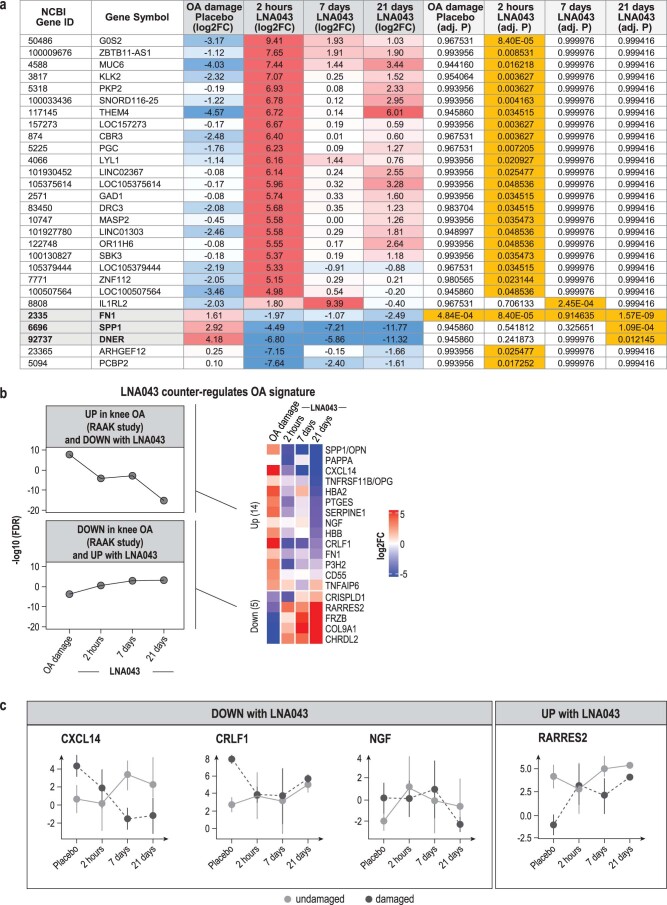
Genes significantly regulated by LNA043 treatment and RAAK OA signature counter-regulated by LNA043. **a**, Log_2_[FC] (log2FC) of genes significantly regulated at either 2 h, 7 or 21 d after LNA043 treatment in damaged OA cartilage compared to placebo (Limma moderated *t*-test, Bejamini and Hochberg, *P* ≤ 0.05). Most-significantly regulated genes are shown in bold; fold upregulation is visualized by red color intensity, fold downregulation by blue color intensity, adjusted *P*-values (adj. P) ≤ 0.05 are shaded in yellow **b**, Temporal counter-regulation of RAAK OA signatures by LNA043. Signatures were generated from the RAAK study published by Ramos *et al*.^38^. The enrichment significance for each geneset, defined as the negative log_10_ of the False Discovery Rate (-log10(FDR)), is plotted over time. Signs depict the direction of enrichment (positive for geneset upregulation and negative for geneset downregulation). Heatmap of the expression profiles of individual genes in the RAAK signatures. OA-associated changes in damaged versus undamaged OA cartilage are reverted by LNA043 treatment across all three timepoints in the FIH study. Downregulated genes, besides *FN1* and *OPN*, encode *TNFRSF11B/OPG* (a subchondral bone remodeling regulator in OA^39,40^), inflammatory mediators like cytokine receptor-like factor 1 (*CRLF1*)^84^ and the middle zone articular cartilage marker C-X-C motif chemokine ligand 14 (*CXCL14*)^85^, as well as nerve growth factor (NGF) the key mediator of pain in OA (reviewed by Dietz *et al*.^63^). Upregulated genes encode cartilage development pathway members whose expression correlates negatively with OA, besides *CHRDL2*, *FRZB* and *COL9A1*, the adipokine chemerin (*RARRES2*)^86^. Data are expressed as log _2_[FC](log2FC). **c**, Temporal expression profiles of selected members of the RAAK signatures in damaged and undamaged cartilage upon LNA043 treatment. Expression levels are expressed as log_2_[CPM] (log2CPM). Data is represented as mean ± s.d.

To further characterize the molecular effects of LNA043, we performed post-hoc global transcriptomics profiling (RNA sequencing (RNA-seq)) on damaged and undamaged cartilage collected from patients at the time of TKR. Transcriptome profiles were generated from three patients treated intra-articularly with a single dose of 20 mg LNA043 2 h, 7 d or 21 d pre-TKR, as well as from seven patients receiving the placebo (Fig. 6b). Statistical modeling was performed to identify OA-responsive (comparing damaged versus undamaged cartilage from placebo-treated patients) and LNA043 treatment-responsive genes (comparing LNA043- versus placebo-treated damaged cartilage). While we could not detect OA-responsive genes (adjusted *P* ≤ 0.05), probably due to the small cohort size, 28 genes were significantly regulated upon LNA043 treatment, most of them responding within the first 2 h (Fig. 6c and Extended Data Fig. 5a). Three of the genes strongly downregulated by LNA043 have been described as potential mediators of OA progression: the very highly expressed fibronectin (*FN1*) and its splice variants^33,34^, osteopontin (*SPP1/OPN*)^35^ and delta/notch-like EGF repeat-containing transmembrane receptor (*DNER*)^36^ (Fig. 6c).

Gene set enrichment analysis (GSEA) was performed leveraging OA-relevant gene sets assembled from data integration initiatives, such as SkeletalVis^37^ and relevant publications. Strikingly, we found several OA signatures to be significantly enriched in our patient damaged cartilage transcriptome, all with concordant regulation directions (for example, the human Research Arthritis and Articular Cartilage (RAAK) knee OA study signatures^38^ (false discovery rate (FDR) = 10^−11^ for the upregulated genes and FDR = 10^−6^ for the downregulated genes; Fig. 6d). This concordance suggests that the observed modest OA gene expression changes in the FIH study could be biologically meaningful.

Importantly, the same OA signatures showed significant reversal upon LNA043 but not placebo treatment: 18 of the 19 genes of the RAAK disease signature were counter-regulated with LNA043 treatment, including *FN1* (Extended Data Fig. 5b,c). Strikingly, Ramos et al.^38^ identified FN1 as the most interconnected protein among these 19 OA proteins, proposing a central role of FN1 in the disease-associated changes. In summary, the data suggest that FN1 plays a key role in both OA pathophysiology and the therapeutic mechanism of action of LNA043. We also uncovered a global trend for a time-dependent counter-regulation of OA genes by LNA043 treatment (Fig. 6e). Interestingly, LNA043 induced transcriptional changes up to 21 d post-treatment, suggesting a prolonged effect of LNA043 in cartilage lasting well beyond the time of LNA043 detection in serum (Extended Data Fig. 3a).

Focusing on the OA transcripts most strongly counter-regulated by LNA043, we observed not only downregulation of the potential OA progression mediators *FN1*, *SPP1/OPN*, *DNER* and osteoprotegerin (*TNFRSF11B/OPG*)^39,40^, but also upregulation of the essential cartilage matrix components matrilin-4 (*MATN4*)^41^ and collagen type IX (*COL9A1*)^42^. Furthermore, LNA043 upregulated expression of the WNT signaling inhibitors *DKK1* and frizzled-related protein (*FRZB*)^43^ and downregulated WNT signaling activators (for example, *WNT16*). LNA043 also upregulated the bone morphogenic protein (BMP) signaling inhibitors chordin-like-2 (*CHRDL2*)^44^ and gremlin-1 (*GREM1*)^45^ and downregulated *BMP* expression (for example, *BMP6*) (Fig. 6e). Inhibition of WNT and BMP signaling pathways has been reported to have cartilage anabolic^46^ and anti-hypertrophic effects^47,48^, respectively. Collectively, these data suggest that LNA043 functions through FN1 and signaling via these OA-relevant pathways.

## Discussion

We describe the discovery and characterization of the ANGPTL3-derivative LNA043, a potent inducer of chondrogenesis and hyaline cartilage regeneration and a novel DMOAD candidate for the intra-articular treatment of OA. To our knowledge, ANGPTL3 has not previously been reported to play a role in chondrogenesis or cartilage regeneration. ANGPTL3 belongs to a family of eight ANGPTL proteins that contain an amino (N)-terminal coiled-coil domain and a C-terminal FBN-like domain^49^. ANGPTL3 is expressed and secreted by the liver and is post-translationally regulated by glycosylation and proteolytic cleavage between the N- and C-terminal domains^50,51^.

The N-terminal domain of ANGPTL3 plays a key role in increasing circulating triglyceride and cholesterol levels^52,53^. In addition, this domain supports expansion and stemness of hematopoietic stem cells in the bone marrow niche^54–57^. Because LNA043 does not contain the N-terminal portion of ANGPTL3, it is not expected to have metabolic or hematopoietic effects, which was confirmed in our FIH study, where no effects on plasma lipid levels or hematology were observed. Unchanged ANGPTL3 serum levels^32^ indicate that LNA043 treatment does not interfere with endogenous ANGPTL3 production or function.

The C-terminal FBN-like domain of ANGPTL3 induces endothelial cell adhesion and angiogenesis through binding to integrin α_v_β_3_ (ref. ^24^). Importantly, we found that LNA043, unlike ANGPTL3, does not induce angiogenesis. Consistently, no local angiogenesis in knee tissues was detected in the FIH study and we observed no interaction between LNA043 and α_v_β_3_ in co-IP studies nor a role for α_v_β_3_ in mediating LNA043 activity in chondrocytes. This suggests that the truncation and point mutation, introduced when deriving LNA043 from ANGPTL3, eliminated the risk of angiogenesis induction.

While chondrogenic activity of ANGPTL3 has not been reported previously, ANGPTL2 is expressed in chondrocytes and accumulates in the surrounding ECM, contributes to chondrocyte differentiation and subsequent endochondral ossification during bone formation^22^ and upregulates the expression of inflammation markers in chondrocytes^58^. ANGPTL4 accumulates in cartilage ECM as well but is induced by hypoxia and promotes cartilage matrix remodeling and erosion^23,59^. Interestingly, we observed that full-length ANGPTL2, ANGPTL3 and ANGPTL4, like LNA043, stimulate secretion of the chondrogenesis inducer DKK1, but also cause cytotoxicity, suggesting that the N-terminal ANGPTL domain may have detrimental effects on chondrocytes.

We demonstrate that LNA043 is a potent inducer of chondrogenesis in primary hMSC pellet culture, where it dose dependently induces expression of the essential cartilage matrix superficial zone protein PRG4 (lubricin)^18,60^ and secretion of the WNT signaling inhibitor and chondrogenesis inducer DKK1 (refs. ^19,43,61,62^). LNA043 also has chondroanabolic effects on mature chondrocytes in human OA cartilage explants where it increases the amounts of PRG4 and the main cartilage matrix component, collagen type II. Furthermore, LNA043 may have chondroprotective effects, as suggested by its suppression of the osteogenic and adipogenic differentiation markers *ALPL* and *LEP*, respectively.

Importantly, our observation that LNA043 partially preserved chondrogenesis and cartilage matrix production and inhibited the expression of inflammatory cytokine IL-6 in the presence of poly(I:C) suggests that LNA043 might be able to regenerate cartilage even in an inflammatory OA milieu. Poly(I:C), a synthetic analog of double-stranded RNA, was used to mimic OA-like conditions, where double-stranded RNA is released from damaged cartilage and promotes cartilage degeneration via the TLR3 pathway^26^. The ability of LNA043 to restore PRG4 secretion by primary human OA chondrocytes after stimulation with the inflammatory cytokines TNF-α + IL-1β further supports LNA043 activity under inflammatory conditions. A clinical study is currently ongoing to confirm LNA043 efficacy in patients with OA and joint inflammation (NCT04814368).

Further investigations are required to assess whether LNA043 also has analgesic activity, as suggested by LNA043 downregulation of nerve growth factor (*NGF*)^63^ in OA cartilage on day 21 (Extended Data Fig. 5b,c). We anticipate improvement of OA pain with LNA043 treatment primarily as a result of cartilage regeneration and recovery of joint homeostasis.

Following confirmation that LNA043 chondrogenesis activity in vitro translates to cartilage regeneration in preclinical models of OA and cartilage injury in vivo and is safe in toxicology studies, an FIH study in patients scheduled for TKR was initiated. LNA043 displayed a favorable safety profile and rapid distribution from the joint to the systemic circulation. Interestingly, we observed that, after a single intra-articular injection, LNA043 penetrates primarily damaged OA cartilage and only the superficial zone of undamaged cartilage. Since the molecular structure and properties of the LNA043 protein do not provide a rationale for this observation, we hypothesize that the degeneration and loosening of the ECM makes damaged OA cartilage more accessible to LNA043 penetration. This suggests that LNA043 may act mainly on areas of damaged cartilage that require repair, and this may contribute to the excellent safety profile of LNA043, which did not induce cartilage overgrowth or ectopic cartilage formation in preclinical safety and efficacy studies.

Although fast clearance of LNA043 from the joint and rapid elimination from the serum after intra-articular injection occurred as expected^64,65^, we observed in post-hoc transcriptomics analyses of cartilage collected at TKR that a single intra-articular injection of LNA043 modifies the expression of genes in damaged cartilage for at least 21 d, indicating that LNA043 elicits long-term pharmacodynamic effects on cartilage-resident cells.

**Extended Data Fig. 6:**
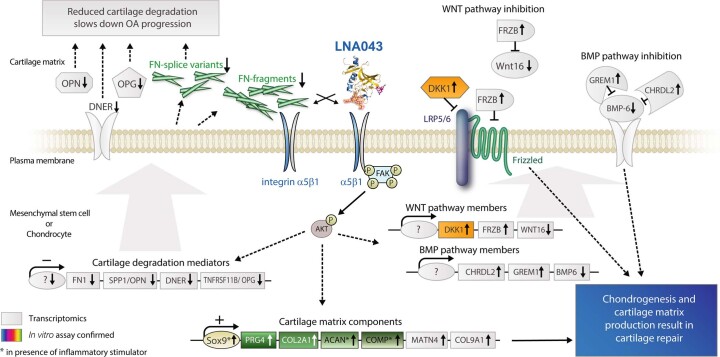
LNA043 MoA hypothesis. LNA043 binding to the FN1 receptor, integrin α_5_β_1_, and intracellular signaling e.g. via FAK and AKT phosphorylation induces transcriptional counter-regulation of OA-relevant gene expression. Specifically, a, up-regulation of essential cartilage matrix components *PRG4*, *COL2A1*, *ACAN*, *COMP*, *MATN4*, *COL9A1* results in cartilage matrix synthesis; b, regulation of genes *DKK1*, *FRZB*, *WNT16* inhibits WNT signaling, which induces chondrogenesis and cartilage regeneration; c, regulation of genes *CHRDL2*, *GREM1*, *BMP6* inhibits BMP signaling, which prevents detrimental chondrocyte hypertrophy and mineralization in OA; d, downregulation of OA progression mediators *OPN*, *DNER*, *OPG* as well as *FN1* splice variants and *FN1*, which in the OA environment is cleaved into cartilage degradation-promoting fragments, reduces cartilage degeneration and progression of OA. Transparent symbols depict transcriptomics results, colored symbols *in vitro* assay-confirmed data.

The transcriptomics results and in vitro assay data allowed us to generate a first LNA043 mechanism of action hypothesis (Extended Data Fig.  6). FN1 seems to play a central role among the LNA043-regulated genes, as it is one of only three genes significantly downregulated by LNA043 for as long as 21 d and is a central interaction partner of the LNA043 counter-regulated OA genes first identified in the RAAK study^38^. FN1 is an adhesive glycoprotein and a fibrillary component of the cartilage ECM that acts as a bridging molecule in matrix assembly and at cell–matrix interfaces^33^. FN1 enhances cartilage repair by activating progenitor cell proliferation, migration and chondrogenic differentiation^66^. However, in OA, FN1 splice variants and fragments are generated that induce cytokine and proteinase expression and thereby establish a degradative and inflammatory feedback loop, which exacerbates OA^33,34^. The observed long-term downregulation of *FN1* in OA cartilage by LNA043 may thus prevent the production of FN1 splice variants and fragments and thereby OA exacerbation.

FN1 exerts its action through binding to integrin α_5_β_1_ receptors expressed on chondrocytes and, even 10-fold higher, on chondrogenic progenitors^66,67^. Integrins mediate interactions between cartilage cells and their surrounding ECM—most notably FN1 and collagen type II—and thereby regulate cell differentiation, proliferation, survival and matrix remodeling and serve as mechano-transducers^27,68^. Importantly, our finding that LNA043 binds recombinant integrin α_5_β_1_ lets us hypothesize that LNA043 exerts at least some of its activities through binding to the FN1 receptor and mimicking the cartilage repair effects of FN1 (Extended Data Fig. 6).

We demonstrate here that LNA043 engagement of integrin α_5_β_1_ mediates secretion of the WNT signaling inhibitor DKK1 in chondrocytes, and that LNA043 treatment upregulates the expression of *DKK1* and *FRZB* (another WNT inhibitor) in human OA cartilage. FRZB functional polymorphisms confer susceptibility to OA and their identification has first suggested a role of the WNT signaling pathway in OA pathogenesis^69,70^. DKK1 inhibits hypertrophic differentiation and MMP expression in chondrocytes^19,45,71^ and reduces cartilage destruction upon overexpression in mice^62^. Consistently, both DKK1 and FRZB expression levels correlate negatively with OA severity^43,61^. Therefore, screening for small-molecule inhibitors of WNT signaling was used to identify the DMOAD candidate lorecivivint^72^, which inhibits WNT signaling at the level of intranuclear kinases^73^. In contrast, LNA043 seems to act upstream by increasing expression of the extracellular WNT signaling inhibitors DKK1 and FRZB and more broadly by also regulating other signaling pathways involved in cartilage regeneration and homeostasis, such as the BMP pathway. LNA043 upregulated expression of the BMP inhibitors *GREM1* and *CHRDL2* in damaged OA cartilage, which inhibit chondrocyte hypertrophy and OA development^44,45,61,74^. It remains to be clarified whether all of the observed transcriptional changes of essential cartilage matrix components, WNT and BMP signaling inhibitors and OA pathogenesis mediators are directly induced by LNA043 binding to α_5_β_1_ (Extended Data Fig. 6) or by regulating each other, as suggested for example by WNT pathway regulation of *OPN*^72^.

In conclusion, the results of our study suggest that LNA043 induces hyaline cartilage repair by mimicking the engagement of FN1 with its integrin α_5_β_1_ receptor on MSCs and chondrocytes, thus counter-regulating the expression of genes that are induced in OA. Further investigations are required to evaluate whether these observed broad effects of LNA043 treatment on OA cartilage are all mediated by LNA043 binding to integrin α_5_β_1_ or whether LNA043 has additional molecular targets. To investigate whether the activities of LNA043 observed in this study translate into a clinical benefit, LNA043 is currently in a phase 2b trial (NCT04864392) in patients with knee OA.

## Methods

### Cell culture for in vitro assays

For 3D pellet culture assays, bone marrow-derived hMSCs from four different donors (Lonza Verviers) were first expanded for two passages in Lonza’s MSCGM BulletKit medium and stored in liquid nitrogen. Cells were further expanded in Dulbecco’s modified Eagle medium (DMEM), 1 g l^−1^ glucose, 10% fetal bovine serum (FBS), 6 mM l-glutamine, 10 mM HEPES, 50 IU ml^−1^ penicillin, 50 μg ml^−1^ streptomycin and 1 ng ml^−1^ human bFGF (R&D Systems). For 3D cultures, passage-6 cells were seeded at 3.5 × 10^5^ cells per well in 96-well V-bottom plates (Costar), sedimented by centrifugation (5 min; 250*g*) and cultured for 4 weeks in DMEM high glucose, 0.125% bovine serum albumin (BSA; Sigma–Aldrich), ITS (6.25 μg ml^−1^ human insulin, 6.25 μg ml^−1^ human transferrin and 6.25 ng ml^−1^ sodium selenite; Roche), 5.3 μg ml^−1^ linoleic acid (Sigma–Aldrich), 50 μg ml^−1^ l-ascorbic acid phosphate (Wako Pure Chemical), 100 ng ml^−1^ dexamethasone (Sigma–Aldrich), 40 µg ml^−1^ proline (Sigma–Aldrich), 100 IU ml^−1^ penicillin and 100 μg ml^−1^ streptomycin, supplemented as indicated with LNA043 (recombinantly expressed in Chinese hamster ovary cells; Novartis) or vehicle control. The medium was changed three times per week.

The human chondrocyte cell line C-28/I2 (licensed from M. Goldring at the Massachusetts General Hospital) was expanded in growth medium containing DMEM/F-12, 10% FCS (Millipore), 50 μg ml^−1^ l-ascorbic acid phosphate, 100 IU ml^−1^ penicillin and 100 μg ml^−1^ streptomycin. To test LNA043, ANGPTL2, ANGPTL3 or ANGPTL4 activity, cells were seeded as a monolayer at 50,000 cells per well (96-well plate format; Costar) and treated immediately for 24 h with LNA043, human ANGPTL2, human ANGPTL3, human ANGPTL4 or vehicle control in 100 μl DMEM/F-12 medium with 1% FCS (Millipore), 50 μg ml^−1^ l-ascorbic acid phosphate, 100 IU ml^−1^ penicillin and 100 μg ml^−1^ streptomycin.

The immortalized human bone marrow-derived mesenchymal stem cells UE7T-13 (JCRB Cell Bank) were expanded in DMEM high glucose, GlutaMAX, sodium pyruvate medium supplemented with 10% FBS (Millipore), 10 mM HEPES, non-essential amino acids, 1 ng ml^−1^ recombinant human bFGF, 100 IU ml^−1^ penicillin and 100 μg ml^−1^ streptomycin. To study LNA043 activity, cells were seeded two-dimensionally at 20,000 cells per well on a 96-well plate (Costar) pre-coated with bovine type I collagen (Viscofan BioEngineering) placed in chondrogenic medium consisting of DMEM high glucose, GlutaMAX, 10 mM HEPES, non-essential amino acids, 0.05% bovine serum albumin (A1595; Sigma–Aldrich), ITS (5 μg ml^−1^ human insulin, 5 μg ml^−1^ human transferrin and 5 ng ml^−1^ selenious acid; Corning Premix), 10 nM dexamethasone (Sigma–Aldrich), 4.7 μg ml^−1^ linoleic acid (Sigma–Aldrich), 100 μM l-ascorbic acid phosphate, 10 ng ml^−1^ recombinant human TGF-β3 (Novartis), 400 ng ml^−1^ human BMP2 (Novartis), 100 IU ml^−1^ penicillin and 100 μg ml^−1^ streptomycin, supplemented as indicated with LNA043, poly(I:C) (Sigma–Aldrich) or vehicle control and grown for the indicated period of time. The medium was changed after 7 d.

Human primary articular chondrocytes were isolated from the femur of three patients with OA (64–82 years old; male) undergoing TKR at the Praxisklinik Rennbahn (Muttenz, Switzerland). All participants provided written informed consent before enrollment in the study and the study was approved by the local medical ethics committee (EKNZ project ID 2020-01812). Sliced cartilage was cut into small pieces and digested at 37 °C under 5% CO_2_ first in 1% pronase (Roche) for 30 min and then in 0.3% type II collagenase (Worthington Biochemical Corporation) for 16 h, based on a modification of the method described by Otero et al.^75^. The isolated chondrocytes were then grown according to Chawla et al.^48^ with the following modifications: straight after isolation, the cells were resuspended and plated in culture dishes at a density of 190,000 cells per cm^2^ in DMEM/F-12, 10% FBS (Millipore), 100 IU ml^−1^ penicillin and 100 μg ml^−1^ streptomycin for 3 d. Following trypsinization, the cells were minimally expanded for 4 d at 10,000 cells per cm^2^ in DMEM high glucose, GlutaMAX, sodium pyruvate medium supplemented with 10% FBS, 0.1 mM non-essential amino acids, 10 mM HEPES, 100 IU ml^−1^ penicillin, 100 μg ml^−1^ streptomycin, 5 ng ml^−1^ human bFGF and 1 ng ml^−1^ TGF-β3 (Novartis). Thereafter, chondrocyte 3D pellets were generated after centrifugation of the cells at 350*g* for 5 min (200,000 cells per pellet) and cultivated in chondrogenic medium containing DMEM high glucose, GlutaMAX, sodium pyruvate medium supplemented with 0.125% BSA (A4919; Sigma–Aldrich), 0.1 mM non-essential amino acids, 10 mM HEPES, 100 IU ml^−1^ penicillin, 100 μg ml^−1^ streptomycin, 10 μg ml^−1^ insulin (Sigma–Aldrich), 5.5 μg ml^−1^ transferrin (Roche), 5.5 ng ml^−1^ Na selenite (Roche), 4.7 μg ml^−1^ linoleic acid (Roche), 0.1 mM l-ascorbic acid phosphate, 0.1 μM dexamethasone (Sigma–Aldrich) and 10 ng ml^−1^ human TGF-β3 (Novartis) for 2 weeks. Thereafter, cells were treated with 1 ng ml^−1^ human recombinant IL-1β (R&D Systems) and 1 ng ml^−1^ human recombinant TNF-α (R&D Systems) for 3 d. The inflammatory insult was subsequently removed and chondrogenic medium (without TGF-β3) supplemented with LNA043 or vehicle was added to the cells for a recovery phase of 7 or 11 d. The medium was changed twice per week.

For the cartilage explant assay, human tibia plateaus were collected from patients with OA undergoing TKR at the Praxisklinik Rennbahn (Muttenz, Switzerland). All participants provided written informed consent before enrollment in the study and the study was approved by the local medical ethics committee (EKNZ project ID 2020-01812). Full-thickness, 4-mm biopsy punches were taken from areas with various degrees of OA-related cartilage degeneration. Each cartilage disk was cut in half. One half was treated with LNA043 at 300 μg ml^−1^ while the other half was treated with vehicle. All explants were cultured in Williams Medium E (PAN Biotech) with 100 IU ml^−1^ penicillin and 100 mg ml^−1^ streptomycin (Gibco) at 37 °C and under 5% CO_2_ for 24 h, after which supernatants were collected for collagen type II and PRG4 ELISAs. A total of 15 cartilage explants were collected from two tibia plateaus from two patients for this study.

The angiogenesis assay was performed at Reaction Biology (Freiburg, Germany) in a modified version of the protocol described by Korff and Augustin^5^. Briefly, human endothelial cell spheroids were prepared as described^76^ by pipetting 400 HUVEC cells in a hanging drop on plastic dishes to allow overnight spheroid aggregation. Some 50 HUVEC spheroids were then seeded in 0.9 ml of a collagen gel and pipetted into individual wells of a 24-well plate to allow polymerization. LNA043, ANGPTL3 or positive controls (VEGF-A and bFGF) were added after 30 min by pipetting 100 μl of a tenfold-concentrated working solution on top of the polymerized gel. The plates were incubated at 37 °C for 24 h and fixed by adding 4% paraformaldehyde (PFA; Carl Roth). Endothelial cell sprouting was quantified by measuring the sprout length and the cumulative sprout length.

### Cell viability and proliferation assay

To determine cell viability, CellTiter-Glo assay (Promega) was performed according to the manufacturer’s instructions on C-28/I2 cells, cultivated immediately after seeding, for 24 h in C-28/I2 test medium supplemented with LNA043. To determine cell proliferation, CellTiter-Glo assay was performed according to the manufacturer’s instruction on UE7T-13 cells, cultivated for 3, 7, 11 and 14 d, in UE7T-13 chondrogenic medium supplemented or not with 0.5 μg ml^−1^ poly(I:C) or 100 or 200 μg ml^−1^ LNA043. Luminescence was measured on a Mithras LB 940 reader.

All cell cultures were performed at 37 °C in a humidified incubator under 5% CO_2_. All cell culture additives were from Life Technologies unless otherwise indicated.

### IHC and Alcian blue staining of in vitro assays

After 3D culture of hMSCs, cell pellets were stored dry at −80 °C before being embedded in optimal cutting temperature matrix, cut into 6-μm cryosections, mounted on SuperFrostPlus slides (631-0108; VWR) and stored back at −80 °C. For immunostaining, cryosections were fixed for 10 min at 4 °C in 1% PFA before a heat-induced epitope retrieval (10 mM citrate buffer; pH 6.0; 95 °C; 10 min) step, followed by permeabilization with 0.05% Triton X-100 and saturation in 10% normal donkey serum (NDS; Sigma–Aldrich) for 1 h. Sections were then incubated for 2 h with mIgG1 anti-lubricin (1:500) monoclonal antibody in 1% NDS, followed by 1 h incubation with Alexa Fluor 488 donkey anti-mIgG1 in 1% NDS. Counterstaining was done with 4′,6-diamidino-2-phenylindole (DAPI) and slides were mounted with Mowiol medium (2.1 M Mowiol (Calbiochem), 2.6 M glycerol (VWR) and 100 mM Tris (pH 8.0)). Negative controls were processed in the same way, but with the corresponding isotype antibody (Supplementary Table 1). Images were prepared with an Olympus VS120 scanner and analyzed with MATLAB (R2015a; MathWorks). Alcian blue staining of cartilage glycosaminoglycans was performed by incubating 4% PFA fixed cells with 1% Alcian Blue solution (Sigma–Aldrich) for 4 h, followed by two washing steps in 3% acetic acid and two washing steps in water.

### ELISA

ELISAs were performed on cell culture supernatants stored at −80 °C. The reagents used are detailed in Supplementary Table 1. Plates were pre-coated overnight at 4 °C with a monoclonal anti-human DKK1 antibody (MSD MULTI-ARRAY High Bind 96-well plates), an anti-human IL-6 antibody (MSD MULTI-ARRAY Standard 96-well plates) or an anti-collagen type II antibody (MSD MULTI-ARRAY Standard 96-well plates), followed by blocking with 1% casein in TBS (Bio-Rad) for 1 h in a ThermoMixer at 450 r.p.m. and then washed four times with 0.5× Tris-buffered saline with 0.1% Tween 20 (TBST; Sigma–Aldrich) at room temperature. Cell supernatants were added in the appropriate wells and incubated at room temperature in a ThermoMixer for 1 h at 450 r.p.m., followed by four washing steps and the addition of biotinylated anti-human DKK1, anti-human IL-6 or anti-collagen type II secondary antibody and further incubation at room temperature in a ThermoMixer for 1 h at 450 r.p.m. After four washing steps, a streptavidin sulfo-TAG solution (0.25 μg ml^−1^; MSD) was added and incubated at room temperature in a ThermoMixer for 30 min at 450 r.p.m. Four additional washing steps were performed before the addition of 2× read buffer (MSD) for detection of the electrochemiluminescence counts with an MSD Sector S 600 reader. For PRG4, MSD MULTI-ARRAY Standard 96-well plates were pre-coated overnight at 4 °C with an anti-human PRG4 antibody, followed by blocking with 1% casein in TBS for 1 h in a ThermoMixer at 450 r.p.m., and then washed three times with 1× TBST at room temperature. Cell supernatants were added in these wells and incubated at room temperature in a ThermoMixer for 2 h at 450 r.p.m., followed by three washing steps and the addition of biotinylated anti-human PRG4 antibody, then further incubation at room temperature in a ThermoMixer for 2 h at 450 r.p.m. The streptavidin sulfo-TAG addition and electrochemiluminescence detection steps were similar to those described above for DKK1, IL-6 and collagen type II ELISA. The COMP and leptin ELISAs were performed according to the manufacturer’s instructions.

### Quantitative reverse transcription PCR-based gene expression assessment

Cell pellets or layers were lyzed in TRIzol before extracting RNA using a Direct-zol-96 RNA kit (R2056; Zymo Research). The RNA concentration and quality were determined using DropSense 96 (PerkinElmer). Total RNA was reverse transcribed with a high-capacity complementary DNA reverse transcription kit according to the manufacturer’s protocol. Gene expression analyses were performed with a QuantStudio 5 Real-Time PCR system or an ABI PRISM 7900HT Sequence Detection System (Thermo Fisher Scientific), for hMSC and UE7T-13 cells, respectively, using TaqMan Universal PCR Master Mix and the following human TaqMan probes: ACAN (Hs00153936_m1), ALPL (Hs01029144_m1), COMP (Hs00164359_m1), IL-6 (Hs00985639_m1), LEP (Hs00174877_m1) and SOX9 (Hs00165814_m1). GUSB (Hs00939627_m1) or HPRT1 (Hs02800695_m1) were used as housekeeping genes for hMSCs and UE7T-13 cells, respectively. All reagents were from Thermo Fisher Scientific, unless otherwise indicated. The relative abundance was calculated according to the formula 2^−[deltaCt (GOI − HKG)]^, where GOI is the gene of interest and HKG is the housekeeping gene.

### Integrin knockdown in C-28/I2 cells

C-28/I2 cells were plated into a T25 flask (Costar) at a density of 4.7 × 10^5^ cells per flask in the growth medium described above. After 24 h, cells were transfected with 30 nM control siRNA (4390843; Ambion), siRNA to integrin α_5_ (siRNA s7547; Ambion), siRNA to integrin α_v_ (s7547; Ambion) or a combination of siRNA to α_5_ and α_v_ using Lipofectamine RNAiMAX according to the manufacturer’s instructions (Invitrogen). After 48 h, cells were detached using Accutase (Invitrogen) and seeded onto 96-well plates at a density of 50,000 cells per well in growth medium containing 1% FCS, supplemented with either LNA043 at the indicated concentrations or vehicle control, and incubated for 24 h. Cell supernatants were analyzed for DKK1 expression by ELISA as described above. In parallel, cells were washed twice with cold PBS and lysed with RIPA buffer (Pierce) containing protease and phosphatase inhibitors (Thermo Fisher Scientific) for 15 min on ice then spun at 4 °C for 10 min at 14,000*g*. Protein concentrations were determined using a BCA Protein Assay Kit (Pierce). Sodium dodecyl sulfate–polyacrylamide gel electrophoresis (SDS-PAGE) sample buffer was added to the samples and equal amounts of proteins were separated using 4–12% SDS-polyacrylamide gels (NuPAGE Novex 4–12% Bis-Tris gels from Invitrogen) and transferred onto nitrocellulose membranes using a Trans-Blot Turbo Transfer System (Bio-Rad). Membranes were blocked in TBST and 5% (wt/vol) milk powder or 5% BSA. Primary and secondary antibodies were incubated in TBST and 5% milk or 5% BSA. Immunoreactive bands were detected by enhanced chemiluminescence using ECL Prime reagent (Amersham) and captured using a FUSION FX (Vilber) system (Supplementary Table 1). The protein band intensity was quantitated by densitometry with Fiji software.

### Co-IP experiments

A total of 200–1,000 ng samples of recombinant human α_5_β_1_ integrin (rhα_5_β_1_; R&D Systems) or recombinant human α_v_β_3_ integrin (rhα_v_β_3_; R&D Systems) were incubated with 200 ng LNA043 in DMEM/F-12 medium (Life Technologies) for 1 h at 37 °C. Mouse monoclonal antibody to human α_5_β_1_, human α_v_β_3_ or mIgG were coupled to magnetic beads according to the manufacturer’s instructions (Invitrogen). Samples containing the LNA043–integrin mixture were added to the magnetic bead–antibody complex for 10 min at room temperature and processed according to the manufacturer’s instructions. Immune complexes were separated by 4–12% SDS-PAGE, transferred onto nitrocellulose membranes and subjected to immunoblotting as described above (Supplementary Table 1).

### SPR analysis of LNA043 binding to integrins

Binding of LNA043 and FN1 to the integrin α_5_β_1_ and of LNA043 and VTN to the integrin α_v_β_3_ was measured on a Biacore T200 instrument. Before the experiments, the integrin analytes (α_5_β_1_ and α_v_β_3_) were re-buffered in running buffer (1× HBS-N, 0.005% Tween 20, 1 mM MgCl_2_, 1 mM CaCl_2_ and 2 mM MnCl_2_) using Amicon spin columns (Millipore). A standard amine coupling procedure was applied to immobilize the molecules on CM5 chips (Cytiva). For this, LNA043 (50 μg ml^−1^) and VTN (5 μg ml^−1^) were diluted in 10 mM sodium acetate buffer at pH 4.5 and FN1 (5 μg ml^−1^) was diluted in 10 mM sodium acetate buffer at pH 4.0. LNA043 was immobilized at a density of ~3,000 resonance units; FN1 and VTN were immobilized at a density of ~500 resonance units on the chip (Supplementary Tables 2 and 3). At first, the chip surface was activated with 1-ethyl-3-(dimethylaminopropyl) carbodiimide/*N*-hydoxysuccinimide and after that the ligands were immobilized on the corresponding flow cells. Remaining active surface groups were subsequently blocked with ethanolamine. Flow cell 1 was left blank to serve as a reference surface. Afterwards, single-cycle kinetic binding data were acquired by five subsequent injections of 1:2 dilution series of analyte (concentration range = 0.6–10 μM) over all flow cells at a flow rate of 30 μl min^−1^ and a temperature of 37 °C. The complexes were allowed to associate and dissociate for 60 and 120 s, respectively. Zero concentration samples (blank run) were measured to allow double referencing during data evaluation. The results were analyzed with the Biacore T200 evaluation software (version 3.0). All raw data were double referenced (blank subtracted and reference flow cell subtracted). The resulting sensorgrams were fitted using a 1:1 binding model to calculate the equilibrium dissociation constants (*K*_d_).

### Rat model of post-traumatic OA

All animal procedures were performed in compliance with Animal Welfare Act regulation 9 CFR (parts 1, 2 and 3) and US regulations as outlined in the *Guide for the Care and Use of Laboratory Animals* and approved by the institutional animal care and use committee. These rat studies were conducted under institutional animal care and use committee protocol 18-455, approved by the Novartis Institutes for BioMedical Research (San Diego, USA) Animal Care and Use Committee.

In all studies, 3- to 4-month-old male LEW/SsNHsd rats (Envigo, USA) were subjected to knee joint surgery to completely sever the medial collateral ligament in combination with inducing a full-thickness tear in the medial meniscus to destabilize the joint so that future weight bearing would lead to rapid degeneration of the articular cartilage. Under isoflurane anesthesia, the medial collateral ligament was severed using a scalpel blade. The scalpel blade was then slipped under the patellar ligament into the synovial space and the pointed tip was used to cut the meniscus. The skin was then closed by 9-mm wound clips (Mikron Precision stainless steel AUTOCLIP clips).

Intra-articular injections were performed under isoflurane anesthesia according to the specified experimental outline. LNA043 clinical service form (CSF) or saline was injected in a volume of 25 μl in the short-term model, and LNA043 CSF or vehicle CSF in 40 μl was injected in the therapeutic model. Injection was through the skin and the patellar ligament into the intra-articular synovial space using a 30G needle attached to a 0.3 ml syringe. At the end of the study, animals were euthanized and joints were harvested, fixed in 10% formalin, decalcified with formic acid and embedded in paraffin before sectioning. Serial step coronal sections were prepared for each knee, each containing two 5-μm serial sections encompassing the entire depth of the joint. Slides were stained with Safranin-O^17^ with adjacent slides that could be used for collagen II/X staining if required (Supplementary Table 1). Using a modified OARSI scoring system (Supplementary Table 4), slides were graded in a blinded manner.

### Minipig cartilage injury model

This study was conducted in the facility of BioAdvice (Denmark) under animal license 2017-15-0201-01187 and in accordance with the Swiss Novartis Animal Care and Use Committee-approved study plan AGR17034, the protocol and facility standard operating procedures.

Thirty-two female minipigs aged 21–26 months at the study start (38.5–55.3 kg) were purchased from Ellegaard Göttingen Minipigs (Denmark) and housed in groups exposed to light cycles of 12 h light and 12 h dark at BioAdvice. Between the day before surgery and 2 weeks after surgery, the minipigs were housed individually.

A full-depth cartilage defect of 6 mm diameter was created surgically in the right trochlea femoris as described^29^. In brief, after the skin incision, the medial border of the patellar tendon was exposed and the joint was entered, taking care not to damage the cartilaginous surfaces. The distal third of the femoral trochlea was exposed and a 6-mm custom-made fenestrated drill guide (Drill Bit; 6.0 mm; length = 195/170 mm, 2-flute for quick coupling; Synthes) was used to remove the cartilage to a depth close to the subchondral bone. The surgical wound was closed in three layers and a spray bandage was applied.

Dosing started 4 weeks post-surgery. Animals were randomly divided into four groups of *n* = 8 and the operated knee was intra-articularly injected with 1.5 ml vehicle CSF (groups 1 and 3) or LNA043 CSF (10 mg ml^−1^) (groups 2 and 4) under anesthesia using a 20G needle. After withdrawal of the needle, the leg was flexed about 20 times (within 1 min) to disperse the injected substance in the joint.

At 6 months (groups 1 and 2) or 12 months (groups 3 and 4), the minipigs were euthanized, synovial fluid was collected and the knee joint, synovial membrane, meniscus and ligaments were observed macroscopically to exclude any inflammatory processes. In addition, a macroscopic evaluation of the knee articular surfaces and surrounding tissues was performed using the ICRS macroscopic cartilage damage score^30^ (Supplementary Table 5). This inverse scoring system ranges from 0 points (excellent cartilage repair) to 20 points (cartilage defect without any repair tissue and extension into the adjacent cartilage). Femoral condyles were explanted from the right knee of each animal, fixed in 4% neutral buffered formalin and sectioned for histological assessment. Cartilage repair was assessed in Safranin-O-stained sections using a modified O’Driscoll score^31^ (Supplementary Table 6).

### FIH study design

The study protocol (NCT02491281) was approved by the Western Institutional Review Board, Puyallup, Washington, USA. Written informed consent was obtained from all participants and the study was performed in accordance with the Declaration of Helsinki. The study started on 16 November 2015 (first visit for first patient) and was completed on 6 March 2018 (last visit for last patient). The last patient was screened on 26 January 2018. The study population comprised male and female patients with primary OA, aged 50–75 years, who were scheduled for TKR, in good general health and on stable medication within the 3 months before. Exclusion criteria were: the use of other investigational drugs within 30 d of enrollment; a history of hypersensitivity to similar drugs; the presence of inflammatory arthropathy, active acute or chronic infection or systemic cartilage disorder; previous cartilage repair surgery; any surgical therapy or local treatment administered intra-articularly into the knee within 2 months before enrollment; a body mass index of >40; the presence of uncontrolled diabetes or hyperthyroidism; large effusion in the knee; corticosteroid use by any route except topical and nasal in the 3 months before enrollment; a history or current diagnosis of cardiovascular disease; a history of malignancy within the past 5 years; pregnant or lactating women; and women of child-bearing potential.

The primary objective of this study was to evaluate the safety and tolerability of LNA043 after one intra-articular injection into the knee. Safety laboratory evaluations included hematology, clinical chemistry and urinalysis. Secondary objectives included evaluation of: the presence and persistence of LNA043 in the joint; LNA043 pharmacokinetics in the serum and concentrations in the synovial fluid; ANGPTL3 levels in the serum and synovial fluid; and immunogenicity in the serum. Exploratory objectives included assessment of the cartilage transcriptome after exposure to LNA043 or placebo, although the analysis was largely not pre-specified.

This was a double-blind study: patients, investigator staff, persons performing the assessments and data analysts remained blinded to the allocation of study treatments. A randomization list was produced under the responsibility of Novartis using a validated system that automates the random assignment of treatment arms to randomization numbers in the specified ratio.

An independent data-monitoring committee was instituted for this study to provide unbiased assessment of potential safety issues. The data-monitoring committee for the study included individuals from the sponsor and from academia with experience in the management of patients with OA.

A total of 30 patients scheduled for TKR were randomized into seven cohorts. Two patients withdrew their consent before dosing and did not receive any study drug. The 28 patients randomized and treated (100%) completed the study. Baseline clinical values and cohort demographics are reported in Fig. 4c.

Seven patients had a protocol deviation related to key procedures not performed as per the protocol. However, none of these patients was excluded from analyses, as none of the protocol deviations was considered to have any relevant impact on safety, pharmacokinetics or biomarker analysis data.

A study protocol summary and results are available at https://www.novctrd.com/ctrdweb/trialresult/trialresults/pdf?trialResultId=17358.

This trial was sponsored by Novartis Pharmaceuticals Corporation.

### FIH study statistical methods

The primary endpoint of this study was safety. No formal sample size calculation was performed and no power evaluation was provided. However, statistical considerations were made related to assessment of adverse events for this study. At a sample size of three patients on active drug per cohort, an adverse event of an underlying occurrence rate of 34% or higher would have ≥70% probability that at least one patient will report an adverse event. If the underlying occurrence rate is 42% or higher then there is ≥80% probability that at least one patient will report an adverse event.

The number and percentage of patients with adverse events were summarized by treatment group and severity. All vital signs, electrocardiogram and laboratory data were summarized using descriptive statistics. Data from patients receiving the placebo were pooled. An unblinded interim analysis was conducted after all patients of cohort 4 had TKR surgery, to select the dose and timepoint before surgery for cohort 5 and 6.

### Key tissue sampling procedures in the FIH study

For every patient, articular cartilage biopsies were harvested during the TKR procedure from macroscopically damaged and undamaged areas (that is, six osteochondral samples (three damaged and three undamaged) and up to three Jamshidi needle biopsies collected from the femoral condyles, tibial plateau and patella). Also, soft tissue samples (anterior cruciate ligament, meniscus and synovial membrane) were obtained. Samples were further processed to assess local drug exposure through IHC staining and bulk RNA-seq analysis using next-generation sequencing methods. Blood samples were collected at study day 1 pre-dose in all cohorts, as well as in cohorts 1–4 and 7 at 15, 120, 240 and 480 min and 4, 8 and 36 d (end of study) post-dose, in cohort 5 at 15, 120 and 240 min and 15 and 29 d (end of study) post-dose and in cohort 6 at 15, 120 and 240 min and 4, 22 and 50 d (end of study) post-dose. Synovial fluid samples were collected at the onset of surgery only in cohorts 1–4.

### LNA043 and ANGPTL3 quantification in the serum and synovial fluid

Concentrations of LNA043 in serum and synovial fluid samples were determined using validated immunocapture liquid chromatography–tandem mass spectrometry assays and anti-ANGPTL3 C-terminal specific antibody (22B16; Supplementary Table 1) with trypsin digestion. The lower limits of quantification were 10 and 20 ng ml^−1^ LNA043 in the serum and synovial fluid, respectively.

Validated sandwich electrochemiluminescence assays were used for quantification of full-length ANGPTL3 protein in human serum and synovial fluid samples utilizing the capture mouse monoclonal anti-ANGPTL3 N-terminal specific antibody (NEG301; Supplementary Table 1) and for detection biotinylated mouse monoclonal anti-ANGPTL3 C-terminal specific antibody (22B16) in combination with Sulfo-Tag-labeled streptavidin. The lower limits of quantification were 2.13 and 2.74 ng ml^−1^ ANGPTL3 in the serum and synovial fluid, respectively.

### Determination of anti-LNA043 antibodies in the serum

The presence of anti-LNA043 antibodies in the serum and their potential cross-reactivity to endogenous ANGPTL3 were determined pre-dose and at the end of the study with a validated bridging electrochemiluminescence assays. In this assay, anti-LNA043 present in the analyzed serum samples formed a bridging complex with biotinylated LNA043 and Sulfo-Tag-labeled LNA043 reagents (Supplementary Table 1).

### IHC staining of cartilage and H-score determination

During the TKR surgery, osteochondral samples consisting of osteochondral fragments were put in 10% neutral buffered formalin for fixation. For femoral, tibial and patella specimen decalcification, the solution was exchanged every 2 h with intermittent checks by needle for the tissue softness during the first 8 h, then samples were left in the Rapid Cal (BBC Biochemical/Fisher Scientific) solution for 68 h (over the weekend), plus another two sets of 2 h periods until the desired softness of each tissue piece was reached. The total time for decalcification was at least 76 h. Decalcified femoral, trochlear tibial and patellar bone/cartilage damaged and undamaged specimens were then bisected, and two formalin-fixed, paraffin-embedded blocks were generated. Formalin-fixed, paraffin-embedded osteochondral specimens were sectioned at 4 μm onto positively charged glass slides. The ANGPTL3 IHC staining protocol is summarized in Supplementary Table 7.

### RNA isolation and RNA-seq

Total RNA was extracted from knee cartilage from TKR resection specimens. The RNA quality was evaluated with an Agilent 2100 Bioanalyzer System using Eukaryote Total RNA Pico chips (Agilent Technologies). The sequencing libraries were prepared following the Pico Input Mammalian protocol of the SMARTer Stranded Total RNA-Seq Kit v2 (Takara Bio). When possible, 250 pg RNA per sample was used as input for the sequencing library preparation. In the case of a limited amount of material, the highest possible amount of the sample was used for sequencing library preparation. Libraries were clustered in a high-output flow cell using a HiSeq PE Cluster Kit v4 on a cBot (Illumina). After cluster generation, the flow cell was loaded onto a HiSeq 2500 system for sequencing using the HiSeq SBS kit v4 (Illumina). DNA was sequenced from both ends (paired end) with a read length of 76 base pairs.

### Transcriptomics data analyses

Sequencing reads were aligned to the ENSEMBL Human Genome 76 reference transcriptome (http://ftp.ensembl.org/pub/grch37/release-76/) using the Bowtie 2 aligner^77^ and gene counts were calculated using HTseq^78^. Statistical analyses were performed in R (https://www.r-project.org). Quality metrics on read duplication, transcript integrity, splice junction saturation and gene body coverage were checked using the RSeQC package^79^, version 2.6.2 (http://rseqc.sourceforge.net/). Counts were log_2_ transformed using voom methods (https://rdrr.io/bioc/limma/man/voom.html) from the limma^78,80^ package. Principal component analysis was performed using prcomp (https://www.rdocumentation.org/packages/stats/versions/3.6.2/topics/prcomp). Statistical differential expression analysis was performed via linear modeling using edgeR^81^ and limma^80^ and packages as available from Bioconductor (https://www.bioconductor.org) after filtering for expressed genes (genes with count per million (CPM) values above 1 in at least three samples). Gender and subject were included as co-variates in the linear model. Due to the observed correlation between the transcriptome complexity and the first principal component (PC1), library complexity was also included as a coefficient. The Benjamini–Hochberg method^82^ for multiple testing correction was applied to both differential expression and GSEA results. Pathway analysis and GSEA were performed using the camera function from the limma package, as described by Wu et al.^83^ and Ritchie et al.^80^, using the ranked moderated *t*-statistics for all contrasts as input and a signature database consisting of expert-curated OA-relevant gene sets from large initiatives such as SkeletalVis (http://phenome.manchester.ac.uk/)^37^ or directly from the literature. For example, the OA RAAK gene sets were curated based on Ramos et al.^38^. Heatmaps were generated using the ComplexHeatmap R package^78^.

Data have been made accessible through the NCBI Gene Expression Omnibus (accession code GSE186220).

### Reporting summary

Further information on research design is available in the Nature Portfolio Reporting Summary linked to this article.

## Online content

Any methods, additional references, Nature Portfolio reporting summaries, source data, extended data, supplementary information, acknowledgements, peer review information; details of author contributions and competing interests; and statements of data and code availability are available at 10.1038/s41591-022-02059-9.

### Supplementary information

Supplementary Information Supplementary Fig. 1 and Tables 1–7.

Reporting Summary

### Source data

Source Data Fig. 2 Western blots.

## Figures and Tables

**Extended Data Table 1:**
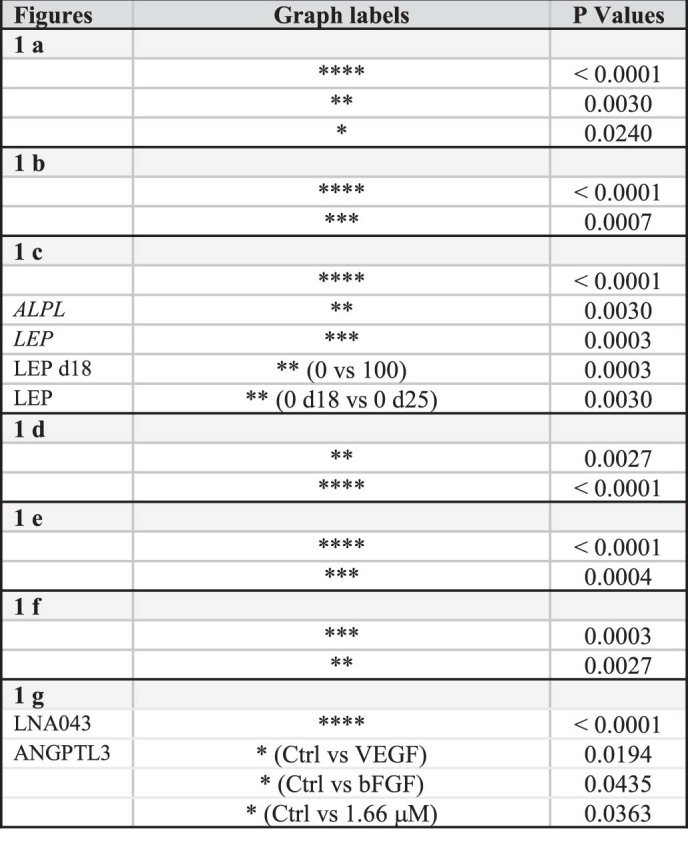
*P* values from Fig. 1

**Extended Data Table 2:**
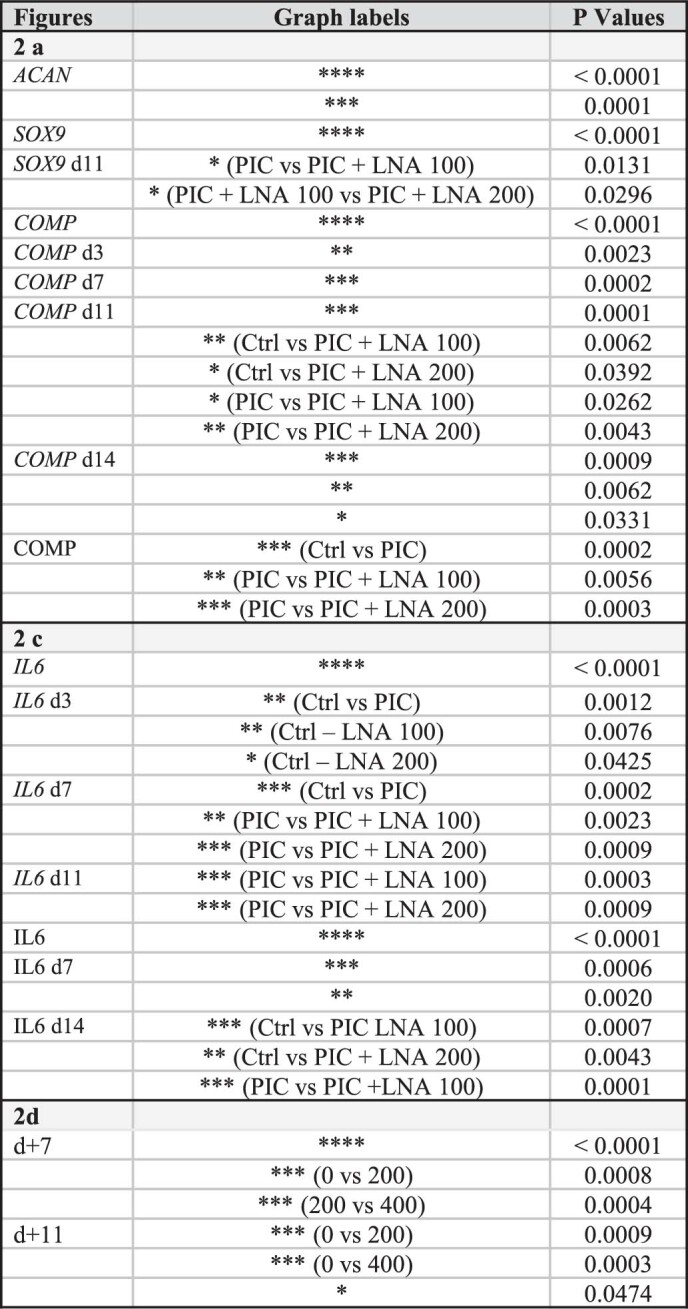
*P* values from Fig. 2

## Data Availability

The bulk RNA-seq data generated during this study have been deposited in the Gene Expression Omnibus with the accession code GSE186220. A summary of the key elements of the CLNA043X2101 trial protocol and results can be found at https://www.novctrd.com/ctrdweb/trialresult/trialresults/pdf?trialResultId=17358. The clinical FIH study datasets generated during and/or analyzed at the end of the present study are not publicly available. Novartis is committed to sharing with qualified external researchers access to patient‐level data and supporting clinical documents from eligible studies. These requests are reviewed and approved on the basis of scientific merit. All data provided are anonymized to respect the privacy of patients who have participated in the trial, in line with applicable laws and regulations. Data may be requested from the corresponding author. Source data are provided with this paper.
