# Supplementary material for: Angiopoietin-like 3-derivative LNA043 for cartilage regeneration in osteoarthritis: a randomized phase 1 trial
Source: Nat Med. 2022 Dec 1;28(12):2633–45. doi: 10.1038/s41591-022-02059-9 (PMC9800282; doi:10.1038/s41591-022-02059-9)
Supplement: Supplementary file 1 — Supplementary Fig. 1 and Tables 1–7. [file 41591_2022_2059_MOESM1_ESM.pdf]

nature medicine

Article https://doi.org/10.1038/s41591-022-02059-9

Angiopoietin-like 3-derivative LNA043 for cartilage regeneration in osteoarthritis: a randomized phase 1 trial

In the format provided by the authors and unedited

LNA043 protein sequence, C-terminus human ANGPTL3 – K423Q (242–460)

```
        10        20        30        40        50        60        70
....|....|....|....|....|....|....|....|....|....|....|....|....|....|
IPAECTTIYNRGEHTSGMYAIRPSNSQVFHVYCDVISGSPWTLIQHRIDGSQNFNETWENYKYGFGRLDG

        80        90       100       110       120       130       140
....|....|....|....|....|....|....|....|....|....|....|....|....|....|
EFWLGLEKIYSIVKQSNYVLRIELEDWKDNKHYIEYSFYLGNHETNYTLHLVAITGNVPNAIPENKDLVF

       150       160       170       180       190       200       210
....|....|....|....|....|....|....|....|....|....|....|....|....|....|
STWDHKAKGHFNCPEGYSGGWWWHDECGENNLNGKYNKPRAQSKPERRRGLSWKSQNGRLYSIKSTKMLI

       219
....|....
HPTDSESFE
```

Supplementary Method Table 1. Reagents used for IHC, ELISA, Immunoblotting, Co-IP, PK, ADA

Description	Company	Catalog number	Dilution / Concentration
IHC of 3D pellets			
Mouse IgG1 anti-lubricin	Millipore	MABT401	2 μg/mL
Mouse IgG1 isotype control	BIORAD	MCA1209	2 μg/mL
AlexaFluor™488 donkey anti-mouse IgG1	Life Technologies	A21202	2 μg/mL
ELISA			
Anti-human DKK1	R&D Systems	MAB10962	2 μg/mL
Biotinylated Anti-human DKK1	R&D Systems	BAF1096	0.25 μg/mL
Anti-human IL-6	R&D Systems	MAB206	1 μg/mL
Biotinylated Anti-human IL6	R&D Systems	BAF206	0.25 μg/mL
R-PLEX human COMP/TPS-5	Mesoscale Discovery	F21UO-3/-8	Manufacturer recommendation
Anti-human Collagen Type II	Chondrex Inc.	7005	3 μg/mL
Biotinylated anti-human Collagen Type II antibody	Chondrex Inc.	7053	1 μg/mL
Anti-human PRG4	R&D Systems	DY9829-05	0.4 μg/mL
Biotinylated Anti-human PRG4	R&D Systems	DY9829-05	0.1 mg/ml PBS, 1% BSA
U-PLEX human Leptin	Mesoscale Discovery	B215Z-3/-8	Manufacturer recommendation
Rat knee IHC			
Anti-type II collagen	Abcam	Ab3092	20 μg/mL
Anti-type X collagen	Abcam	Ab58632	50 μg/mL
Anti-mouse 488	Life Technologies	A-11099	20 μg/mL
Anti-rabbit 594	Life Technologies	A-11005	20 μg/mL
DAPI	Sigma	D8417	2 μg/mL
Immunoblotting			
GAPDH (6C5), Mouse mAb	Ambion	AM4300, RRID:AB437392	0.08 μg/mL
Integrin α5 (D7B7G) Rabbit mAb	Cell Signaling Technology	98204	0.1 μg/mL
Integrin αV (D2N5H) Rabbit mAb	Cell Signaling Technology	60896	0.1 μg/mL
Co-IP			
Monoclonal antibody to α5β1	Millipore	MAB1999	1 μg/IP
Monoclonal antibody to αVβ3	Millipore	MAB1976	1 μg/IP
Mab to hANGPTL3	LSBio	LS-C340259	0.5 μg/mL
PK			
Biotinylated monoclonal anti-ANGPTL3 C-terminus-specific	Novartis Pharma AG	22B16	2.10 mg/mL
Monoclonal anti-ANGPTL3 N-terminus-specific	Novartis Pharma AG	NEG301	2.36 mg/mL
ADA			
Biotinylated LNA043	Novartis Pharma AG	NA	1.98 mg/mL
Sulfo-TAG LNA043	Novartis Pharma AG	NA	2.05 mg/mL
Biotinylated AngPTL4	Novartis Pharma AG	NA	50.1 mg/mL
Sulfo-TAG AngPTL4	Novartis Pharma AG	NA	56.6 mg/mL
monoclonal anti-ANGPTL3 C-terminus-specific	Novartis Pharma AG	22B16	2.10 mg/mL
Human Anti-AngPTL4 antibody	R&D Systems	AF3485	200 mg/mL

ANGPTL3, Angiopoietin-like 3; ANGPTL4, Angiopoietin-like 4; Co-IP, Co-immunoprecipitation; DKK1, Dickkopf-1; IgG, immunoglobin G; IHC, immunohistochemistry; IL6, interleukin 6; mAbs, monoclonal antibodies; PK, Pharmacokinetics; ADA, Anti-Drug-Antibodies.

Supplementary Method Table 2. Reagents for SPR experiments

Material	Vendor	Catalog number
Series S CM5 sensor chip	Cytiva	29-1496-03
10 mM acetate buffer pH 4.0	Cytiva	BR100349
10 mM acetate buffer pH 4.5	Cytiva	BR100350
50 mM NaOH	Cytiva	BR100358
EDC	Fluka	3450
NHS	Fluka	56480
Ethanolamine	Fluka	2400
HBS-N 10x (0.1 M HEPES, 1.5 M NaCl pH 7.4)	Cytiva	BR100670
Tween-20	VWR	437082Q
2 M $MgCl_2$ solution	Sigma-Aldrich	68475-100ML-F
1 M $MnCl_2$ aqueous solution	Alfa Aesar	J63150
$CaCl_2$	Sigma-Aldrich	746495-500G
Amicon spin columns	Millipore	UFC503024

Supplementary Method Table 3. Sample list for SPR experiments

Integrin	Vendor	Catalog number
α5β1	R&D Systems	3230-A5-050
αVβ3	R&D Systems	3050-AV-050
LNA043	Novartis Clinical Service Form (CSF)	
human fibronectin	Novartis Pharma AG	
human vitronectin	Sigma	SRP3186

Supplementary Method Table 4. Modified OARSI cartilage scoring system

<table>
<tr><td>Score of 1</td><td>• Cell loss
• OR loss of Safranin-O intensity
• OR fibrillation on the tibial surface</td><td rowspan="4">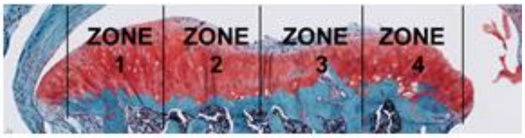

The sum of all 4 zones is calculated (maximum damage score = 16)</td></tr>
<tr><td>Score of 2</td><td>One feature from score 1 plus
• Loss of SafO intensity in deep zone
• OR osteoblast infiltration</td></tr>
<tr><td>Score of 3</td><td>• Complete cartilage ECM loss through deep zone</td></tr>
<tr><td>Score of 4</td><td>• Full thickness lesion</td></tr>
</table>

Modified from Gerwin *et al*. (2010)[28]

Supplementary Method Table 5. ICRS macroscopic cartilage damage score

Parameter	Item	Points
Color of the repair tissue	Hyaline or white	0
	Predominantly white (>50%)	1
	Predominantly translucent (>50%)	2
	Translucent	3
	No repair tissue	4
Presence of blood vessels in the repair tissue	No	0
	Less than 25% of the repair tissue	1
	25-50% of the repair tissue	2
	50-75% of the repair tissue	3
	More than 75% of the repair tissue	4
Surface of the repair tissue	Smooth, homogeneous	0
	Smooth, heterogeneous	1
	Fibrillated	2
	Incomplete new repair tissue	3
	No repair tissue	4
Filling of the defect	In level with adjacent cartilage	0
	>50% repair of defect depth or hypertrophy	1
	<50% repair of defect depth	2
	0% repair of defect depth	3
	Subchondral bone damage	4
Degeneration of adjacent articular cartilage	Normal	0
	Cracks and/or fibrillations in integration zone	1
	Diffuse osteoarthritic changes	2
	Extension of the defect into the adjacent cartilage	3
	Subchondral bone damage	4
**Total points**		**20**

Source: Goebel *et al*. (2012)[30]

Supplementary Method Table 6. Modified O'Driscoll cartilage repair score

Characteristic	Score
**1- Nature of predominant tissue**	
- Hyaline cartilage	4
- Mostly hyaline cartilage	3
- Mixed hyaline and fibrocartilage	2
- Mostly fibrocartilage	1
- Some fibrocartilage, most non-chondrocytic cells	0
**2- Structural characteristics**	
**A- Surface regularity**	
- Smooth and intact	3
- Superficial horizontal lamination	2
- Fissures 25-100% of the cartilage thickness	1
- Severe disruption, including fibrillation	0
**B- Structural integrity, homogeneity**	
- Normal	2
- Slight disruption, including cysts	1
- Severe disruption, including fibrillation	0
**C- Thickness**	
- 100% of normal adjacent cartilage	2
- 50-100% of normal cartilage	1
- 0-50% of normal cartilage	0
**D- Bonding to adjacent cartilage**	
- Bonded at both ends of graft	2
- Bonded at one end or partially at both ends	1
- Not bonded	0
**3- Freedom from cellular changes of degeneration**	
**A- Hypocellularity**	
- Normal cellularity	3
- Slight hypocellularity	2
- Moderate hypocellularity or hypercellularity	1
- Severe hypocellularity of hypercellularity	0
**B- Chondrocyte clustering**	
- No clusters	2
- <25% of the cells	1
- 25-100% of the cells	0
**4- Safranin-O staining**	
- Normal or near normal	3
- Moderate reduction ≤ 25%	2
- Low staining 25 to up to 75% reduction	1
- None >75%	0
**Maximal score**	**21**

Source: O'Driscoll *et al*. (2001)[31]

Supplementary Method Table 7. ANGPTL3 IHC protocol

**Antibody**	ANGPTL3, mouse monoclonal clone 5E6
**Vendor**	LSBio (cat# LS-C340259)
**Antigen retrieval**	30 min steamer, Tris based buffer, pH 10
**Antibody dilution**	1:150 (6.7 µg/mL)
**Detection system**	MACH4 Mouse probe, MACH4 HRP Polymer (Biocare)
**Chromogen**	DAB
**IHC Run Control**	Normal Human liver (all runs)
