# Supplementary material for: Angiopoietin-like 3-derivative LNA043 for cartilage regeneration in osteoarthritis: a randomized phase 1 trial
Source: Nat Med. 2022 Dec 1;28(12):2633–45. doi: 10.1038/s41591-022-02059-9 (PMC9800282; doi:10.1038/s41591-022-02059-9)
Supplement: Supplementary file 2 — Reporting Summary [file 41591_2022_2059_MOESM2_ESM.pdf]

nature portfolio

Corresponding author(s): Nicole Gerwin

Last updated by author(s): Sep 14, 2022

Reporting Summary

Nature Portfolio wishes to improve the reproducibility of the work that we publish. This form provides structure for consistency and transparency in reporting. For further information on Nature Portfolio policies, see our Editorial Policies and the Editorial Policy Checklist.

Statistics

For all statistical analyses, confirm that the following items are present in the figure legend, table legend, main text, or Methods section.

n/a	Confirmed	
☐	☒	The exact sample size (*n*) for each experimental group/condition, given as a discrete number and unit of measurement
☐	☒	A statement on whether measurements were taken from distinct samples or whether the same sample was measured repeatedly
☐	☒	The statistical test(s) used AND whether they are one- or two-sided<br>*Only common tests should be described solely by name; describe more complex techniques in the Methods section.*
☐	☒	A description of all covariates tested
☐	☒	A description of any assumptions or corrections, such as tests of normality and adjustment for multiple comparisons
☐	☒	A full description of the statistical parameters including central tendency (e.g. means) or other basic estimates (e.g. regression coefficient) AND variation (e.g. standard deviation) or associated estimates of uncertainty (e.g. confidence intervals)
☐	☒	For null hypothesis testing, the test statistic (e.g. *F*, *t*, *r*) with confidence intervals, effect sizes, degrees of freedom and *P* value noted<br>*Give P values as exact values whenever suitable.*
☒	☐	For Bayesian analysis, information on the choice of priors and Markov chain Monte Carlo settings
☒	☐	For hierarchical and complex designs, identification of the appropriate level for tests and full reporting of outcomes
☒	☐	Estimates of effect sizes (e.g. Cohen's *d*, Pearson's *r*), indicating how they were calculated

Our web collection on statistics for biologists contains articles on many of the points above.

Software and code

Policy information about availability of computer code

Data collection	No software was used to collect the data in this study
Data analysis	Sequencing reads were aligned to the ENSEBML Human Genome [76] reference transcriptome (http://ftp.ensembl.org/pub/grch37/release-76/) using the BOWTIE2 aligner [77] and gene counts were calculated using HTseq version 0.6.1p1 [78]. Statistical analyses were performed in R (https://www.r-project.org) version 4.0.4.<br>Quality metrics on read duplication, transcript integrity, splice junction saturation and gene body coverage were checked using the RSeQC package [79], version 2.6.2 (http://rseqc.sourceforge.net/).<br>Counts were Log2 transformed using ''voom" method s (https://rdrr.io/bioc/limma/man/voom.html) from the limma [78,80] package version 3.46.0. Principal component analysis (PCA) was performed using "prcomp" (https://www.rdocumentation.org/packages/stats/versions/3.6.2/topics/prcomp). Statistical differential expression analysis was performed via linear modeling using edgeR [81] and limma [80] and packages as available from Bioconductor (https://www.bioconductor.org) after filtering for expressed genes (genes with count per million (CPM) values above 1 in at least 3 samples).<br>Gender and subject were included as co-variates in the linear model. Due to the observed correlation between the transcriptome complexity and the first principal component (PC1), library complexity was also included as an additional coefficient. The Benjamini and Hochberg method [82] for multiple testing correction was applied to both differential expression and GSEA results.<br>Pathway and GSEA were performed using the "camera" function from the limma package as described by Wu et al., 2012 [83] and Ritchie et al., 2015 [80] using the ranked moderated t-statistics for all contrasts as input.<br>Heatmaps were generated using the ComplexHeatmap R package [78].<br><br>Literature:<br>77. Langmead, B. & Salzberg, S.L. Fast gapped-read alignment with Bowtie 2. Nat Methods 9, 357-359 (2012).<br>78. Anders, S., Pyl, P.T. & Huber, W. HTSeq--a Python framework to work with high-throughput sequencing data. Bioinformatics 31, 166-169 (2015).

79. Wang, L., Wang, S. & Li, W. RSeQC: quality control of RNA-seq experiments. Bioinformatics 28, 2184-2185 (2012).
80. Ritchie, M.E., et al. limma powers differential expression analyses for RNA-sequencing and microarray studies. Nucleic Acids Res 43, e47 (2015).
81. Robinson, M.D., McCarthy, D.J. & Smyth, G.K. edgeR: a Bioconductor package for differential expression analysis of digital gene expression data. Bioinformatics 26, 139-140 (2010).
82. Benjamini, Y. & Hochberg, Y. Controlling the False Discovery Rate: A Practical and Powerful Approach to Multiple Testing. Journal of the Royal Statistical Society. Series B (Methodological) 57, 289-300 (1995).
83. Wu, D. & Smyth, G.K. Camera: a competitive gene set test accounting for inter-gene correlation. Nucleic Acids Res 40, e133 (2012).

For manuscripts utilizing custom algorithms or software that are central to the research but not yet described in published literature, software must be made available to editors and reviewers. We strongly encourage code deposition in a community repository (e.g. GitHub). See the Nature Portfolio guidelines for submitting code & software for further information.

Data

Policy information about availability of data

All manuscripts must include a data availability statement. This statement should provide the following information, where applicable:

- Accession codes, unique identifiers, or web links for publicly available datasets
- A description of any restrictions on data availability
- For clinical datasets or third party data, please ensure that the statement adheres to our policy

A summary of key elements of the CLNA043X2101 trial protocol and results can be found at https://www.novctrd.com/ctrdweb/trialresult/trialresults/pdf?trialResultId=17358.
The clinical FIH study data sets generated during and/or analyzed at the end of the present study are not publicly available. Novartis is committed to sharing with qualified external researchers access to patient-level data and supporting clinical documents from eligible studies. These requests are reviewed and approved on the basis of scientific merit. All data provided is anonymized to respect the privacy of patients who have participated in the trial in line with applicable laws and regulations. The data may be requested from the corresponding author of the manuscript

RNASeq data has been made accessible through the gene expression Omnibus: GEO Accession number GSE186220.

For pathway analysis and GESEA, we used a signature a database consisting of expert curated OA relevant genesets from large initiatives such as SkeletalVis (SkeletalVis (ncl.ac.uk) or directly from literature. i.e.

- Soul, J., Hardingham, TE., Boot-Handford, RP. & Schwartz, JM. SkeletalVis: An exploration and meta-analysis data portal of cross-species skeletal transcriptomics data, Bioinformatics. https://doi.org/10.1093/bioinformatics/bty947 (2018)
- Ramos, YF., den Hollander, W., Bovée, JV., Bomer, N. et al. Genes involved in the osteoarthritis process identified through genome wide expression analysis in articular cartilage; the RAAK study. Gene Expression Omnibus: GSE57218.
- Soul, J., Anand, S., Schwartz, JM. Boot-Handford, RP. & Hardingham, TE. RNA-Seq analysis of human intact and damaged osteoarthrtitic cartilage following total knee replacement. ArrayExpress: E-MTAB-4304.
- Fisch, KM., Gamini, R., Alvarez-Garcia, O., Akagi, R., Saito, M., Muramatsu, Y. Sasho, T., Su, AI. & Lotz, MK. Identification of transcription factors responsible for dysregulated networks in human osteoarthritis cartilage by global gene expression analysis. Gene Expression Omnibus: GSE114007.
- Stelcer, E., Kulcenty, K., Rucinski, M., Jopek, K., Trzeciak, T., Richter, M. & Suchorska, WM. Forced differentiation in vitro leads to stress-induced activation of DNA damage response in hiPSC-derived chondrocyte-like cells. Gene Expression Omnibus: GSE108035.
- Wei, X. & Li, Y. Expression data of LncRNA from the rat (Sprague-Dawley) knee articular cartilage at different developmental stages. Gene Expression Omnibus: GSE66554.
- Rai, MF., Patra, D., Sandell, LJ. & Brophy, RH. Transcriptome Analysis of Human Injured Meniscus Reveals a Distinct Phenotype of Meniscus Degeneration with Aging. Gene Expression Omnibus: GSE45233.

Field-specific reporting

Please select the one below that is the best fit for your research. If you are not sure, read the appropriate sections before making your selection.

☒ Life sciences ☐ Behavioural & social sciences ☐ Ecological, evolutionary & environmental sciences

For a reference copy of the document with all sections, see nature.com/documents/nr-reporting-summary-flat.pdf

Life sciences study design

All studies must disclose on these points even when the disclosure is negative.

Sample size

- FIH study: Safety analysis was the primary endpoint in this study. No formal sample size calculation was performed, and no power evaluation provided. However, statistical considerations were made related to assessment of AEs for this study. At a sample size of 3 patients on active drug per cohort, an AE of an underlying occurrence rate of 34% or higher would have ≥ 70% probability that at least one patient will report an AE. If the underlying occurrence rate is 42% or higher then there is ≥ 80% probability that at least one patient will report an AE.

- Rat OA models: Sample sizes of n=8-10 were calculated for treatment groups and n=3-5 for naive rats using Systat version 13 software with data from previous in-house experiments on severity and variability of the OA phenotype and expected treatment effect in the short-term and therapeutic models.
Minipig cartilage injury study: Sample sizes of n=8 were calculated using Systat version 13 software with previous data from Dr. Mainil-Varlet and AGINKO on the variability of cartilage defects and treatment effects in this minipig cartilage injury model.

- in vitro experiments: No sample size calculations were performed. Sample size was chosen based on the many experiments performed in

	each experimental model. These experiments have shown that a biological replicate of 3 was sufficient since the reproducibility between the replicates was good.
Data exclusions	- FIH study: Two patients, randomized but not dosed, were excluded from all analysis sets. No patient was excluded from the ones who received treatment.<br>- Preclinical studies: No data were excluded in either of the three studies, however 5 rats died during the short-term rat OA study for reasons unrelated to LNA043 treatment.
Replication	- FIH study: no replication was performed, because the clinical safety endpoint was met and permitted to progress to a Phase 2a clinical trial.<br>- Short-term rat OA study: not repeated, since the results provided sufficient confidence to progress to evaluation in the therapeutic rat OA model.<br>- Therapeutic rat OA study was repeated two times and comparable results were obtained.<br>- Minipig cartilage injury study was not repeated since the results provided sufficient confidence of LNA043 efficacy to progress to clinical studies.<br>-In vitro experiments: Repeats (minimum 3) were performed in most in vitro experiments as detailed in the respective Figure legends. The only exceptions were experiments shown in Fig. 1e, where the ANGPTLs were tested only in 2 experiments, because of the large concentrations needed; in Fig. 1f, only one experiment was performed, because it was performed on the reviewer's request and no time for repetition; in Fig 2a and 2c, the gene expression read out was measured and reproduced in only 2 experiments, while three other read outs (ELISA, Alcian blue and cellular morphology) were measured and reproduced in 3 experiments; in Extended Fig. 1b, one experiment (with biological triplicate) has been performed and another experiment with slightly different cell seeding density confirming the data of the initial experiment. Exemplary experiments are shown in all Figures except for Fig. 3a, where data from several experiments were pooled, as indicated in the Figure Legend.
Randomization	- FIH study: 30 patients were randomized into 7 planned cohorts. In each cohort, 4 patients were randomized 3:1 (active:placebo).<br>- Preclinical studies: in all 3 preclinical studies animals were randomly assigned to treatment groups.
Blinding	- FIH study: double blinded.<br>- Preclinical studies: In all 3 studies histopathology scoring and macroscopic scoring were performed blinded to treatment.

Reporting for specific materials, systems and methods

We require information from authors about some types of materials, experimental systems and methods used in many studies. Here, indicate whether each material, system or method listed is relevant to your study. If you are not sure if a list item applies to your research, read the appropriate section before selecting a response.

Materials & experimental systems

n/a	Involved in the study
☐	☒ Antibodies
☐	☒ Eukaryotic cell lines
☒	☐ Palaeontology and archaeology
☐	☒ Animals and other organisms
☐	☒ Human research participants
☐	☒ Clinical data
☒	☐ Dual use research of concern

Methods

n/a	Involved in the study
☒	☐ ChIP-seq
☒	☐ Flow cytometry
☒	☐ MRI-based neuroimaging

Antibodies

Antibodies used	Please find all antibodies used in Supplementary Methods Tables 1 and 7
Validation	- ANGPTL3, mouse monoclonal clone 5E6 from LSBio (cat# LS-C340259) allowed to detect LNA043 in human cartilage sections by immunohistochemistry.<br>- Biotinylated monoclonal anti-ANGPTL3 C-terminus-specific Ab (22B16) and monoclonal anti-ANGPTL3 N-terminus-specific Ab (NEG301) were identified, produced and validated at Novartis Pharma AG and allowed LNA043 and ANGPTL3 detection, respectively, in human pharmacokinetic analyses. Validation experiments of monoclonal anti-ANGPTL3 NEG301 confirmed that it recognizes the N-terminus of ANGPTL-3 and validation of monoclonal anti-ANGPTL3 22B16 confirmed that it is C-terminus-specific.<br>- Monoclonal antibodies to alpha5beta1 (MAB1999) and to alphaVbeta3 (MAB1976) from Millipore, as well as Mab to hANGPTL3 (LS-C340259) from LSBio were successfully used in co-immunoprecipitation experiments with a human chondrocyte cell line.<br>- Mouse mAb to GAPDH (6C5) from Ambion, rabbit mAb to integrin alpha5 (D7B7G) and rabbit mAb to integrin alphaV (D2N5H) from Cell Signaling Technology were successfully used in Western blot experiments with a human chondrocyte cell line.<br>- Mouse mAb anti-lubricin (MABT401) from Millipore, Anti-human DKK1 mAb (MAB10962), biotinylated Anti-human DKK1 (BAF1096), Anti-human IL-6 (MAB206) and biotinylated Anti-human IL6 (BAF206), anti-human PRG4, biotinylated anti-human PRG4 all from R&D Systems, anti-human Collagen Type II and biotinylated anti-human Collagen Type II antibody from Chondrex Inc. have been used successfully for ELISAs with human cell culture supernatants.<br>- Mouse IgG1 anti-lubricin (MABT401) from Millipore was successfully used for immunohistochemistry of cell pellets.<br>- Anti-type II collagen Ab (Ab3092) and Anti-type X collagen Ab (Ab58632) were successfully used for immunohistochemistry in rat knee joint sections.

Eukaryotic cell lines

Policy information about cell lines

Cell line source(s)	C-28/I2: licensed from Dr Mary Goldring, Massachusetts General Hospital, Boston, USA, who established this line. There is no commercial source for this line.<br>UE7T-13: JCRB Cell Bank
Authentication	None of the cell line used were authenticated by us
Mycoplasma contamination	All cell lines were tested negative for mycoplasma contamination
Commonly misidentified lines<br>(See ICLAC register)	None found in the ICLAC register

Animals and other organisms

Policy information about studies involving animals; ARRIVE guidelines recommended for reporting animal research

Laboratory animals	Male LEW/SsNHsd rats from ENVIGO, Indianapolis, USA were 3-4 months old at study start.<br>Female minipigs from Ellegaard Göttingen Minipigs (A/S, Dalmose, Denmark) were 21-26 months old at study start.
Wild animals	No wild animals were used in the study
Field-collected samples	No flied-collected samples were used in the study
Ethics oversight	- All rat procedures were in compliance with Animal Welfare Act regulations 9 CRF Part 1, 2, and 3 and US regulations as outlined in the Guide for the Care and Use of Laboratory Animals and approved by the institutional animal care and use committee (IACUC). The rat studies were conducted under IACUC protocol # 18-455 approved by the Genomics Institute of the Novartis Research Foundation Animal Care and Use Committee.<br>- The minipig study was conducted in the facility of BioAdvice (Ølstykke, Denmark) under animal license 2017-15-0201-01187 and in accordance with the Swiss Novartis Animal Care and Use Committee-approved study plan no. AGR17034, the protocol, and with facility Standard Operating Procedures.

Note that full information on the approval of the study protocol must also be provided in the manuscript.

Human research participants

Policy information about studies involving human research participants

Population characteristics	This study included male and female patients aged 50 to 75 years of age diagnosed with primary OA of the knee for whom a TKR was planned. Patients had to be in good health, with a health status acceptable for TKR surgery and on stable medications within 3 months prior to enrollment. Informed consent was obtained from all participants.<br><br>Key exclusion criteria included presence of inflammatory arthropathy, active acute or chronic infection, or systemic cartilage disorder, prior cartilage repair surgery, any surgical therapy or local treatment to the knee within 2 months prior to enrollment, large effusion in the knee and corticosteroid use by any route except topical and nasal in the 3 months prior to enrollment. A history of any venous thromboembolism, transient ischemic attack, stroke, intracranial hemorrhage, arteriovenous malformation, vasculitis, bleeding disorder, coagulation disorders or screening blood tests that indicate altered coagulability (e.g. platelet count, activated partial thromboplastin time (aPTT), prothrombin time (PT)/international normalized ratio (INR) tests) led to exclusion. Women of child-bearing potential were also excluded.<br><br>Patients were recruited and data collected at the Investigational site (Arizona Research Center). Patients were compensated for travel costs, including parking, and meals.<br><br>Human primary articular chondrocytes and human tibia plateaus for the cartilage explant assay were isolated from the femur of three OA patients (64-82 years old, male) undergoing total knee replacement at the Praxisklinik Rennbahn (Muttenz, Switzerland).
Recruitment	Eligible patients were included in the study after providing written (witnessed, where required by law or regulation), IRB approved informed consent.<br>Informed consent was obtained before conducting any study-specific procedures (i.e. all of the procedures described in the protocol). The process of obtaining informed consent was documented in the patient source documents. The date of signing of informed consent (and withdrawal, if later withdrawn) was documented in the case report/record form (CRF).<br>Novartis provided Investigators a proposed informed consent form that complies with the ICH Good clinical practice (GCP) guideline and regulatory requirements and was considered appropriate for this study. Any changes to the proposed consent form suggested by the Investigator was agreed to by Novartis before submission to the IRB.
Ethics oversight	WIRB (Western Institutional Review Board); Puyallup, WA 98374-2115 USA; Local Medical Ethics Committee (EKNZ Project-ID 2020-01812), 4132 Muttenz, Switzerland.

Note that full information on the approval of the study protocol must also be provided in the manuscript.

Clinical data

Policy information about clinical studies

All manuscripts should comply with the ICMJE guidelines for publication of clinical research and a completed CONSORT checklist must be included with all submissions.

Clinical trial registration	NCT02491281
Study protocol	A study protocol summary and results are available here: https://www.novctrd.com/ctrdweb/trialresult/trialresults/pdf?trialResultId=17358. A redacted study protocol is enclosed to this submission.
Data collection	The study consisted of an up to 7-day screening period, an up to 7-day baseline period, one single i.a. injection one week (cohort 1 to 4 and 7), 2 hours (cohort 5), and 3 weeks (cohort 6), prior to TKR surgery.<br>Safety follow-up visits or calls were done after dosing and surgery respectively. In case of safety concerns, the patient were asked to return to the hospital for a safety control visit. An End of Study (EoS) visit occurred approximately 28-days after the TKR surgery for all cohorts. Recruitment started on Nov 16th 2015 and ended on Jan 30th 2018. Study completion date was Mar 6th, 2018.
Outcomes	The primary objective was to evaluate the safety and tolerability of LNA043 after one intra-articular (i.a.) injection into the knee of osteoarthritis (OA) patients scheduled for total knee replacement (TKR).<br>Secondary objectives included evaluation of the joint presence and persistence of LNA043 within the cartilage knee tissue, LNA043 pharmacokinetics in serum and concentration in synovial fluid, ANGPTL3 levels in serum and the synovial fluid, and immunogenicity in serum.<br><br>Criteria for evaluation were as follows:<br><br>Safety: Safety assessments consisted of collecting all AEs, SAEs, with their severity and relationship to study drug, physical examinations, vital signs, height and weight, laboratory evaluations (hematology, blood chemistry and urinalysis), ECG, pregnancy and assessments of fertility, immunogenicity and Knee injury and Osteoarthritis Outcome Score (KOOS).<br><br>Pharmacokinetics: Pharmacokinetic parameters of Cmax, Tmax, AUClast were determined in serum using the actual recorded sampling times and non-compartmental methods with Phoenix WinNonlin (Version 6.2 or higher). The linear trapezoidal rule was used for AUC calculations.
