# Supplementary material for: Angiopoietin-like 3-derivative LNA043 for cartilage regeneration in osteoarthritis: a randomized phase 1 trial
Source: Nat Med. 2022 Dec 1;28(12):2633–45. doi: 10.1038/s41591-022-02059-9 (PMC9800282; doi:10.1038/s41591-022-02059-9)

Fig. 3a_WB aV and a5

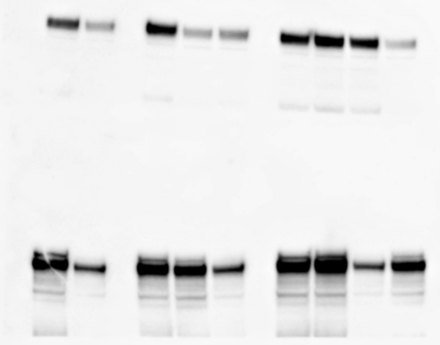

Fig. 3a_WB aV and a5_short exposure

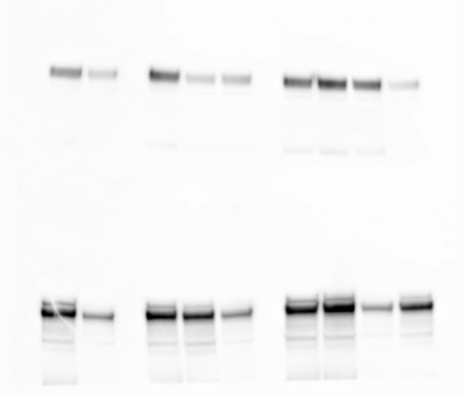

Fig. 3a_WB GAPDH for aV and a5

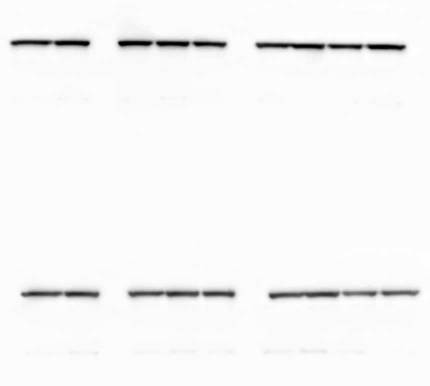

Fig 3b_WB a5 IP

WB aV IP

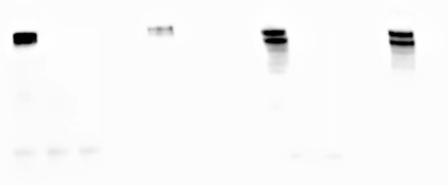

Fig 3b_LNA IP_5sec

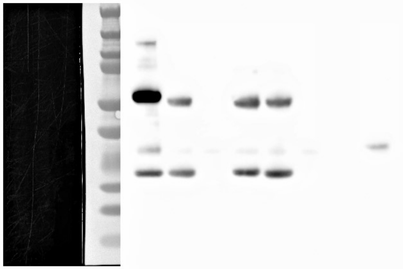

Supplement: Supplementary file 3 — Western blots. [file 41591_2022_2059_MOESM3_ESM.pdf]
